# Next-Generation Artificial Intelligence Strategies for Mechanistic Cancer Target Discovery and Drug Development: A State-of-the-Art Review

**DOI:** 10.3390/ijms27094028

**Published:** 2026-04-30

**Authors:** Muhammad Sohail Khan, Muhammad Saeed, Muhammad Arham, Imran Zafar, Majid Hussian, Adil Jamal, Muhammad Usman, Fayez Saeed Bahwerth, Gabsik Yang, Ki Sung Kang

**Affiliations:** 1 College of Korean Medicine, Gachon University, 1342 Seongnamdaero, Seongnam 13120, Republic of Korea; sohail@gachon.ac.kr; 2 Department of Information Technology, Faculty of Computer Sciences, Lahore Garrison University, Lahore 54000, Pakistan; msaeed4771@gmail.com; 3 Department of Artificial Intelligence, Faculty of Information Technology, The University of Faisalabad (TUF), Faisalabad 38000, Pakistan; drmuhammadarham4@gmail.com; 4 Department of Biochemistry and Biotechnology, The University of Faisalabad (TUF), Faisalabad 38000, Pakistan; bioinfo.pk@gmail.com (I.Z.); adiljamalcemb@gmail.com (A.J.); 5 Department of Computer Science, The University of Faisalabad (TUF), Faisalabad 38000, Pakistan; majidhussain1976@tuf.edu.pk (M.H.); muhammadusman7075717@gmail.com (M.U.); 6 Hera General Hospital, Makkah 21955, Saudi Arabia; fayez-bahwerth@hotmail.com

**Keywords:** artificial intelligence, cancer target discovery, mechanistic modeling, drug development, multi-omics, synthetic lethality, precision oncology

## Abstract

Artificial intelligence (AI) is increasingly used in cancer research, enabling integrative analysis of complex biomedical data to identify actionable therapeutic vulnerabilities. This review specifically examines how AI advances mechanistic cancer target discovery and translational drug development, focusing on: (1) the processing of large-scale genomics, transcriptomics, proteomics, metabolomics, single-cell profiling, spatial, and clinical datasets using machine learning (ML) and deep learning (DL) algorithms; (2) the identification of candidate biomarkers, driver genes, dysregulated pathways, tumor dependencies, and molecular targets that traditional methods often miss; (3) the integration of multi-omics data, network biology, causal inference, and systems-level modeling to refine mechanistic understanding of cancer progression and separate functional driver events from passengers; and (4) applications in drug development, including virtual screening, molecular modeling, structure-informed target validation, drug repurposing, synthetic lethality prediction, and de novo drug design, which collectively may enhance early-stage drug discovery efficiency. The review underscores that AI serves as both a predictive tool and a platform for linking molecular mechanisms to hypothesis generation, target prioritization, and rational treatment design. Challenges such as data heterogeneity, algorithmic bias, interpretability, reproducibility, regulatory requirements, and patient privacy must be addressed for robust translation and clinical use. Future directions may focus on hybrid approaches that integrate causal modeling, explainable AI, multimodal data, and experimental validation to yield mechanistically grounded, clinically actionable insights. AI-driven approaches ultimately aim to accelerate mechanism-based cancer target discovery and enable more precise, biologically informed anticancer therapies.

## 1. Introduction

Cancer remains a leading cause of global morbidity and mortality, defined by extreme biological complexity, interpatient heterogeneity, and continuous evolutionary dynamics. Tumor initiation, progression, metastasis, and therapeutic resistance arise from multilayered alterations spanning genomic to microenvironmental scales, integrated through highly interconnected regulatory networks. This complexity obscures the true functional drivers, as many observed alterations represent context-dependent or passenger events. Moreover, intratumoral heterogeneity, temporal evolution, and adaptive resistance critically hinder the identification of robust, clinically actionable targets that deliver durable therapeutic benefit [1,2].

Recent advances in high-throughput and multi-omics profiling—including genomics, transcriptomics, proteomics, metabolomics, single-cell, and spatial technologies—have enabled unprecedented resolution of tumor biology. These technologies reveal drivers, pathway dysregulation, cell states, and microenvironmental interactions. However, these datasets are high-dimensional, heterogeneous, noisy, and platform-dependent. Integrating and interpreting them remains challenging. Despite the descriptive power of multi-omics data, this approach alone is insufficient for reliably prioritizing actionable targets or guiding translational therapeutic development [3,4].

Artificial intelligence (AI) is best understood as a tool, not a standalone solution, for investigating complex cancer datasets and generating testable hypotheses. AI excels at recognizing patterns, integrating multiple data types, and prioritizing potential targets. Machine learning and deep learning methods find hidden links in extensive biological and chemical data. This facilitates biomarker discovery, subtype classification, drug–target prediction, and lead prioritization [5]. Coupled with biological insight and laboratory validation, AI sharpens the search for actionable cancer drivers. It is important to note that AI output remains predictive and hypothesis-generating, rather than definitive or conclusive for mechanisms or clinical applications [6,7].

Conventional AI is limited by poor mechanistic interpretability, primarily because it captures statistical associations rather than causal biology. In contrast, biologically informed frameworks—such as graph-based models, explainable AI, and causal inference—leverage pathway structure, molecular interactions, and disease context. This improves the prioritization of synergistic drug combinations. These approaches help identify therapeutic pairs and generate hypotheses about pathway co-targeting and resistance vulnerabilities. However, current evidence is mainly computational or preclinical. Therefore, AI-derived predictions serve as tools for hypothesis generation and prioritization, not as validated indicators of clinical synergy or causal mechanisms [1,8].

Integrating multi-omics data with systems biology and graph-based learning enables the identification and ranking of candidate therapeutic targets, tumor-selective antigens, and dysregulated pathways. This integration is key to advancing precision oncology and developing next-generation therapeutics. Integrative AI frameworks can accelerate the development of advanced modalities, such as antibody–drug conjugates (ADCs), by prioritizing targets and guiding structure-informed optimization. For instance, AI-based structural models can enhance antibody affinity, stability, and developability. Generative modeling approaches can inform the design of linker-payload combinations, thereby improving efficacy and reducing off-target toxicity [3,9].

Digital twins and patient-specific modeling frameworks have emerged as tools for simulating treatment response, supporting stratification, and informing adaptive development strategies. Their translational maturity is variable and context-dependent [10]. AI approaches are also used for target-informed drug repurposing and therapeutic design by integrating molecular profiles, pharmacological data, and biological network information. In this review, these tools are discussed mainly in the context of supporting target identification, functional interpretation, and therapeutic hypothesis generation.

AI-driven approaches are increasingly used in drug repurposing and de novo discovery to identify novel target compound associations and therapeutic indications. Integrating multi-omics, bioinformatics, network pharmacology, and molecular dynamics enables systematic characterization of drug–target interactions and prioritization of candidates. Although these frameworks accelerate early-stage discovery, they remain sensitive to data quality, model assumptions, and computational complexity. Rigorous biological validation is required [11,12]. More broadly, combining omics, network analysis, and AI is essential for a systems-level characterization of tumor biology. This enables precise identification of therapeutic vulnerabilities [12,13].

This review examines how advances in next-generation AI are advancing mechanistic cancer target discovery and translational drug development. Unlike broad overviews of AI in oncology, it highlights the role of AI in uncovering biologically meaningful cancer drivers, pathway dependencies, synthetic lethal interactions, and actionable therapeutic vulnerabilities. The review focuses on mechanistically informed and explainable AI, multi-omics integration, network biology, causal inference, and advanced computational modeling. It also shows how these approaches support target prioritization, therapeutic design, target-informed drug repurposing, rational combination strategies, and early translational development. By narrowing the scope to these areas, the review offers a focused, biologically grounded framework that links cancer biology and AI-driven therapeutic innovation.

## 2. AI Foundations for Mechanistic Cancer Target Discovery & Modern Biomedical Research

AI is an important computational framework in cancer research. It can analyze large, heterogeneous, and high-dimensional biomedical datasets. These datasets, derived from next-generation sequencing, multi-omics profiling, and related molecular technologies, offer valuable insights into oncological disease biology. However, their complexity limits interpretation by conventional methods. AI addresses these limitations by identifying biologically meaningful patterns, integrating diverse molecular data, and generating predictive models that support the mechanistic interpretation of cancer processes [14,15]. Notably, the term AI encompasses several computational paradigms, each with distinct purposes, interpretive strengths, and limitations. To provide context, the following section briefly clarifies these paradigms before discussing their unique roles in cancer research.

### 2.1. Conceptual Definitions and Analytical Scope of AI Approaches

Artificial intelligence (AI) in cancer research involves a broad set of computational approaches. These methods differ in architecture, analytical purpose, data requirements, and interpretive depth. Specifically, “conventional AI” or “machine learning (ML)” refers to classical supervised and unsupervised learning, which are used for prediction, classification, clustering, and ranking, working primarily with structured or labeled datasets. Common approaches include logistic regression, support vector machines, random forests, and gradient boosting [16,17,18]. Clustering methods are also widely applied. These applications span cancer subtype classification, biomarker discovery, survival prediction, and treatment-response modeling. While these methods are valuable in biomedical research, their outputs are often association-based, providing limited causal or mechanistic interpretability unless used with additional biological constraints.

Deep learning (DL) is a more advanced subset of AI. It uses multilayer neural-network architectures to learn hierarchical feature representations from high-dimensional data, such as histopathology images, radiologic scans, genomic signals, and transcriptomic matrices [19,20]. In contrast, mechanistic or biologically informed AI refers to approaches that use prior biological knowledge, drawing from sources such as signaling pathways, gene-regulatory networks, protein–protein interaction maps, perturbation data, structural biology, or causal modeling. By leveraging this information, these methods aim to improve biological relevance and target prioritization. More recently, foundation models and generative AI systems are also emerging; these large pre-trained models enable multimodal learning, molecular generation, biomedical language inference, and therapeutic hypothesis generation [21,22,23]. Despite the promise of these advanced methods, important limitations persist, including incomplete external validation, domain shift, reduced explainability, and uncertain translational generalizability. It is important to view these AI categories as complementary, not interchangeable, because each offers distinct strengths and limitations for mechanistic cancer target discovery and drug development.

**Figure 1:**
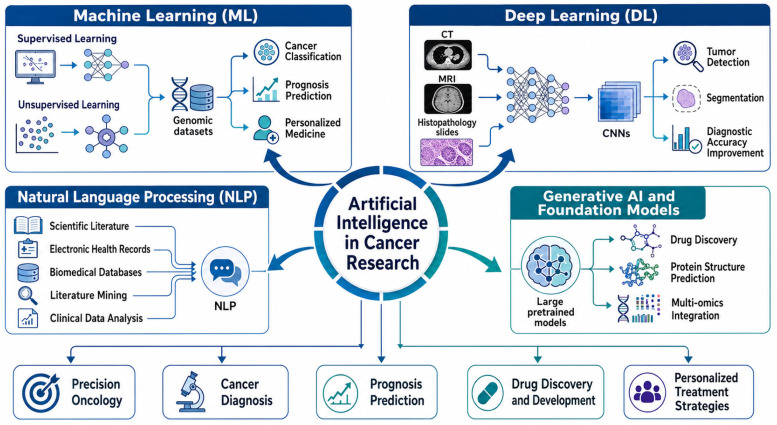
Core artificial intelligence techniques (ML, DL, NLP, and generative AI) and their roles in biomedical and oncology research.

In cancer research, AI can integrate and analyze various biomedical data types. These include genomic sequences, transcriptomic and proteomic data, radiological and imaging data, histopathology data, and clinical data. By integrating such heterogeneous datasets, scientists gain a more detailed view of tumor biology, disease progression, and therapeutic response. This comprehensive perspective enables integrative models to support many applications, such as early cancer diagnosis, tumor profiling, prognosis, biomarker identification, drug discovery, and personalized therapy. Furthermore, as shown in Figure 1, several AI methodologies form the computational basis for these advances in oncology, with key methods including ML, DL, natural language processing (NLP), and generative AI models [16,17].

ML is one of the most widely used AI methods in biomedical research. It is central to cancer data analysis, particularly for prediction, classification, clustering, and biomarker prioritization. In biomedical contexts, these methods are commonly divided into supervised learning and unsupervised learning. ML models are trained on large datasets that capture patterns and relationships. Predictive and classification tasks are performed using learned representations. Supervised learning models use labeled datasets where the outcome is known. They are applied to predictive tasks such as cancer subtype classification, survival prediction, and treatment response modeling. Conversely, unsupervised learning models process unlabeled datasets to uncover latent patterns, groups, or molecular subtypes in complex biological data. ML techniques that analyze genomic and transcriptomic data help identify biomarkers of tumor progression and therapeutic response. This enables patient stratification and the formulation of individualized treatment plans [18,19].

ML, especially DL, has widened the range of analysis in biomedical studies. While many conventional ML approaches rely more on manually engineered features, DL algorithms can automatically extract hierarchical representations from raw or minimally processed data. Convolutional neural networks (CNNs), as one of the most popular DL models, have shown good results in medical imaging analysis. For example, these models can analyze CT, MRI, PET, and digital histopathology images to detect tumors, segment them, and enhance diagnostic accuracy. Thus, DL-based imaging analysis complements radiological and pathological assessments through automated recognition of subtle morphological patterns associated with malignant transformation. This, in turn, may contribute to earlier detection and improved clinical evaluation [20,21].

NLP is another valuable AI technology that enables analysis of large textual data, such as scientific works, clinical records, and biomedical databases. Specifically, NLP methods allow computers to automatically extract information from biomedical literature, electronic medical records, and research archives. Furthermore, these algorithms can discover links among genes, proteins, diseases, drugs, and biological pathways mentioned in scientific literature, as well as identify relationships among these entities. In cancer research, NLP tools support literature mining, clinical data analysis, and knowledge discovery, which helps researchers quickly synthesize existing information and generate new hypotheses for translational research. Additionally, NLP systems can support clinicians by analyzing patient records to reveal patterns related to treatment outcomes and disease progression [22,23].

Recent advances in generative AI and foundation models have broadened the ability to model biological systems of interest. Specifically, these models learn large-scale representations of genes, proteins, and molecular interactions within various biomedical datasets. As a result, they are useful in protein structure prediction, drug-target interaction modeling, and therapeutic discovery. In addition, foundation models trained on multi-omics data can now integrate genomic, transcriptomic, proteomic, and metabolomic information. This integration enhances our understanding of cancer biology and supports the discovery of new therapeutic targets [24,25]. More broadly, the convergence of ML, DL, NLP, and generative AI has improved data analysis, diagnostic modeling, disease prediction, and drug discovery in biomedical applications. Consequently, this progress has made AI more relevant in precision oncology and translational cancer biology [26,27].

### 2.2. AI Frameworks Supporting Mechanistic Target Discovery and Therapeutic Prioritization

Besides basic AI procedures, more sophisticated computational models are increasingly used in mechanistic cancer target discovery. These include multimodal AI, graph-based learning, and knowledge graph-based models. Such methods allow the integration of heterogeneous biological datasets, such as genomic, transcriptomic, proteomic, molecular interaction, and selected clinical data. This integration helps determine complex relationships among genes, signaling pathways, drugs, and disease phenotypes. Instead of acting as generic predictive models, these AI models are especially useful in revealing targetable, biologically significant dependencies, pathway dysregulations, and molecular vulnerabilities in cancer.

Multimodal AI models are especially useful when two or more data layers (such as genomic, proteomic, or imaging data) must be integrated. They help describe tumor biology in more detail and enhance target prioritization. Network-conscious AI, such as graph neural networks (which process data structured as nodes and edges) and knowledge graphs (which organize information as interconnected entities), learns and predicts the structure of biological systems. These approaches identify functional relationships between cancer genes, pathways, and therapeutic drugs, and find mechanistically significant points of intervention.

**Figure 2:**
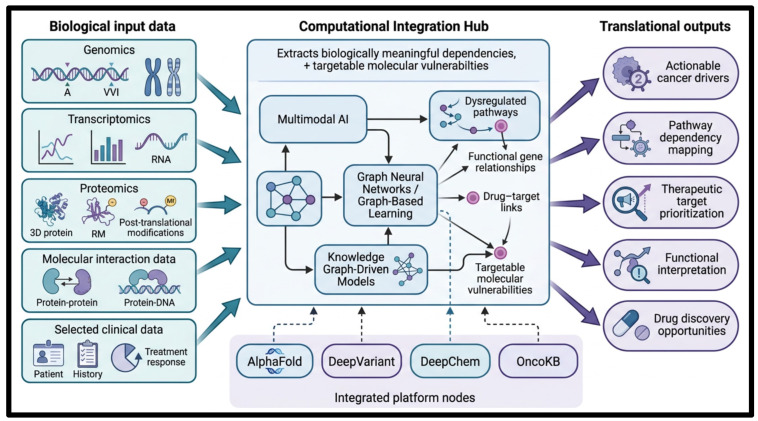
Advanced AI Frameworks for Mechanistic Cancer Target Discovery and Translational Drug Development.

These complementary methodologies are supported by specially designed computational platforms and resources, as shown in Figure 2. The most relevant to this review are AlphaFold for predicting protein structure, DeepVariant for interpreting genomic variants, DeepChem for AI-based drug discovery, and OncoKB for integrating clinically and biologically relevant oncology knowledge. Such tools are most useful when they lead to target identification.

Combined, these AI frameworks form the computational foundation for shifting from descriptive molecular profiling to mechanism-guided target prioritization and translational drug development. Although these methods have broader oncology applications, this review focuses on their use in identifying actionable drivers of cancer, dependencies in pathways, and therapeutic opportunities for drug discovery [28,29].

### 2.3. Integration of Artificial Intelligence Technologies in Precision Oncology

A mix of AI methods has led to better computer models for precision oncology. These models look at molecular, imaging, and clinical data to sort tumors, find biomarkers, predict outcomes, and evaluate treatment responses. They use classic machine learning, deep neural networks, transformer models, graph models, and models that combine different data types. AI’s practical use now also includes imaging, digital pathology, natural language processing, and drug discovery. AI methods such as virtual screening, molecular docking, toxicity predictions, and combining many types of data have improved how therapies are chosen and our understanding of cancer biology. Table 1 lists AI models, platforms, uses, advantages, and limits. Still, concerns remain around data quality, interpretability, privacy, and bias [30,31].

## 3. Artificial Intelligence-Driven Decoding of Cancer Biology and Precision Oncology

**Figure 3:**
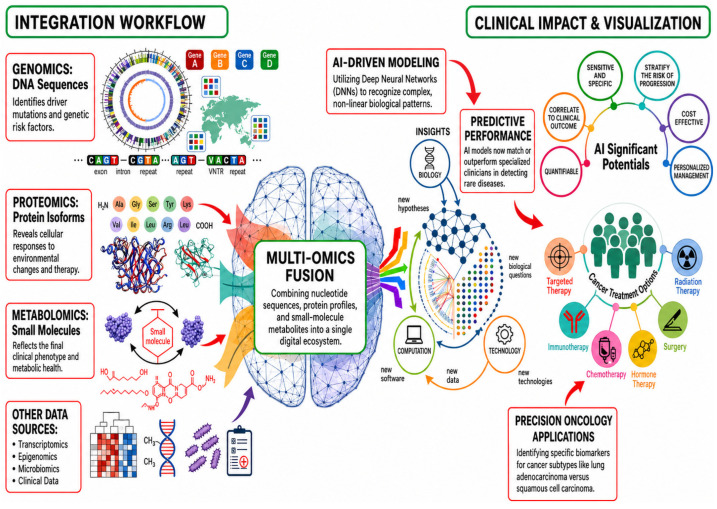
Workflow shows AI-driven integration of multi-omics data (genomics, proteomics, and metabolomics) for biomarker discovery, predictive modeling, and precision oncology applications in cancer research.

Artificial intelligence (AI) is increasingly used to analyze the complexity of big data in cancer at the molecular and cellular level, mainly to support biological understanding of tumors [42]. Using large datasets from genomics, transcriptomics, proteomics, metabolomics, and single-cell studies, AI methods such as machine learning and deep learning combine these datasets. This integration uncovers critical patterns, pathway changes, tumor differences, and possible biomarkers related to cancer progression [11,43,44]. Figure 3 illustrates how linking genomics, proteomics, and metabolomics with computational tools clarifies molecular relationships across biological levels. These methods help prioritize mechanistic targets even if they are not directly for clinical use. Despite challenges with different dataset types, explainability, reproducibility, and validation, these approaches provide an important basis for precision oncology and cancer mechanism research [45,46].

### 3.1. AI-Enabled Cancer Genomics and Tumor Heterogeneity as a Basis for Mechanistic Target Discovery

Genome alterations are significant contributors to cancer development and growth. Mutations in oncogenes, tumor suppressor genes, and DNA repair pathways impair normal cell regulation. These changes promote malignant transformation. Large-scale cancer genome projects, such as The Cancer Genome Atlas (TCGA), have produced extensive data sets. These include somatic mutations, copy number changes, gene expression, and epigenetic modifications across cancer types. As a result, these data provide a solid basis for computational and AI-based analyses in cancer biology research [42].

The application of AI has enhanced the capacity to examine these data sets. It helps determine genomic markers related to tumor growth and progression [31]. Specifically, machine learning methods are used to distinguish driver mutations, which contribute to tumor growth, from passenger alterations with limited biological impact. By analyzing genomic variation across large patient cohorts, AI models can help identify important genes, regulatory nodes, and dysregulated signaling pathways in tumor development and progression [47,48]. This section explains how such analyses help us understand cancer diversity and genetic complexity. These insights then support frameworks that set research priorities [47,48]. AI also assists in selecting features and building models to find biomarkers of cancer subtypes and tumor progression [49]. The main point is how these features help with target ranking and biological understanding, not just broad diagnostic or prognostic use.

DL models find nonlinear links between genomic, transcriptomic, and proteomic signals and certain clinical traits. For example, these models tie molecular profiles to survival, treatment response, and disease recurrence [50,51]. In a mechanistic context, they uncover key relationships, like those between changed pathways, tumor status, and possible points for intervention. Recent studies show AI can blend multi-omics data to model tumor biology as a whole [52]. But cancer cell heterogeneity driven by selective pressures and tumor microenvironments remains a concern, often leading to different treatment responses and drug resistance. AI tools can identify molecular subtypes, predict how tumors evolve, and describe tumor-environment interactions [53]. As Table 2 shows, these studies do more than classify; they help find subtype drivers, resistance pathways, and therapy vulnerabilities based on context.

Advances in single-cell sequencing are transforming tumor heterogeneity research by enabling high-resolution analysis of individual tumor cells. To differentiate cellular subpopulations in complex tumor ecosystems, clustering, dimension-reduction, and graph-learning techniques such as autoencoders and graph neural networks can be used with AI. As a result, these techniques can trace the evolution of tumors, profile cancer stem-like populations, and describe interactions between malignant and immune cells within the tumor microenvironment [53]. In the context of mechanistic target discovery, such analyses are especially useful, as they identify rare resistant cell states, lineage-specific dependencies, and microenvironment-driven signaling programs that bulk datasets often miss. Meanwhile, AI technologies can also connect spatial transcriptomics to histopathology. This integration enables characterization of spatially resolved tumor heterogeneity and tissue organization [54]. Using normal pathology images, these multimodal structures deduce both molecular and cellular patterns, allowing scalable computation of tumor architecture. By integrating genomic, single-cell, spatial, and morphological data, AI may bridge molecular characterization with actionable target hypotheses and therapeutic priorities. Despite challenges such as data integration, model interpretability, reproducibility, and clinical translation [55], these methods form a fundamental basis for mechanism-informed target prioritization and translational cancer drug development.

**Table 2:** AI in cancer genomics and tumor heterogeneity.

No.	Cancer Type	Main AI Method(s)	Data Types	Tumor Heterogeneity/Genomics Focus	Key Finding/Unique Contribution	Main Limitation/Challenge	Clinical or Research Relevance	Citation
1	Esophageal cancer	ML, DL, AI-guided multi-omics integration	Genomics, epigenomics, transcriptomics, proteomics, metabolomics, single-cell, and spatial data	Multi-layer tumor heterogeneity across cellular, genetic, and phenotypic levels	Explains how AI can integrate multi-omics to resolve esophageal tumor heterogeneity and support precision oncology workflows	Integration of highly heterogeneous omics layers remains difficult	Useful framework for biomarker discovery and patient-specific stratification	[56]
2	Breast cancer	Self-supervised DL	H&E histology + spatial omics for training	Tumor microenvironment (TME) heterogeneity	Reports prediction of immune and stromal cell states from routine H&E slides alone, with an AUROC of roughly 0.88–0.97	Depends on spatial-omics-labeled training data; early-stage evidence	Promising low-cost route for TME profiling without full molecular assays	[54]
3	Multiple cancers	ML, DL	Genomics, transcriptomics, proteomics, radiomics, and pathology images	Cross-platform precision oncology	Summarizes how AI extracts latent patterns from multi-modal cancer data for diagnosis, prognosis, and treatment-response prediction	Data quality and cross-cohort heterogeneity	Broad precision-medicine overview linking AI to clinical oncology	[31]
4	Multiple cancers	AI across translational oncology	Multi-omics, single-cell, spatial profiling, clinical data	Translational integration of tumor biology with AI	Highlights AI for target discovery, biomarker identification, patient stratification, and therapy-response prediction	Reproducibility, interpretability, workflow integration	Strong translational perspective for precision oncology	[52]
5	Multiple cancers	Filter, wrapper, embedded feature-selection methods with ML	High-dimensional omics datasets	Tumor subtype classification	Shows feature selection is central for reducing dimensionality and improving interpretability in subtype classification.	Overfitting and instability across datasets	Better biomarker selection for subtype diagnosis	[49]
6	Multiple cancers	AI-driven multi-omics language models/DL	Genomics, transcriptomics, multi-omics	Cancer heterogeneity representation learning	Reviews emerging language-model-style frameworks for integrating omics and improving stratification and drug-response prediction	Standardization and evaluation of these models are still immature	Important future direction for foundation-model oncology	[57]
7	Multiple cancers	ML, DL for multi-omics integration	Genomic, epigenomic, transcriptomic, proteomic, metabolomic data	Early detection, diagnosis, prognosis, treatment	Summarizes how multi-omics AI can link biological mechanisms of heterogeneity to clinical tasks across the cancer-care pathway	Data harmonization and ethical issues	Good umbrella reference for AI-enabled cancer research pipelines	[58]
8	Colorectal cancer	AI-enabled single-cell and spatial transcriptomic analysis	scRNA-seq, spatial transcriptomics	CRC cellular composition and spatial heterogeneity	Explains how AI helps decode cellular interactions and spatial organization in colorectal cancer	Processing and biological interpretation remain hard	Supports precision exploration of CRC heterogeneity	[53]
9	Multiple cancers	Predictive modeling, radiogenomics AI	Radiology + genomic phenotypes	Imaging-linked molecular heterogeneity	Reviews how AI-based radiogenomics can link imaging phenotypes to genomic states, prognosis, and recurrence risk	Difficult fusion of imaging and genomics; standardization issues	Important for non-invasive precision oncology	[59]
10	Breast cancer	Single-cell analytics + interaction modeling	Single-cell RNA-seq breast tumor atlas	Heterogeneity of epithelial–immune interactions	Built a large single-cell breast tumor atlas and derived InteractPrint, which predicts immunotherapy response across breast cancer subtypes	Needs validation across broader clinical cohorts	Strong example of AI/omics translating heterogeneity into response prediction	[60]
11	Breast cancer	DL	Histopathology slides + spatial transcriptomics for training	Spatial gene-expression heterogeneity	Predicts spatial gene expression directly from H&E and reportedly outperforms earlier ST predictors on validation data	Preprint; not yet the final peer-reviewed journal version of the result shown	Could scale spatial profiling to much larger cohorts	[61]
12	Triple-negative breast cancer	Radiomic/radiogenomic modeling	Imaging + molecular data	Intertumoral and peritumoral heterogeneity	Identified radiomic features reflecting peritumoral heterogeneity associated with immune suppression and metabolic reprogramming in TNBC	Generalization across scanners and cohorts can be difficult	Useful for non-invasive risk stratification	[62]
13	Non-small cell lung cancer (NSCLC)	Transformer + graph variational autoencoder	Spatial transcriptomics + morphology images	Tumoral niche heterogeneity	Introduces a framework to identify and characterize tumor niches by combining spatial transcriptomics with morphology	Computational complexity and external validation remain concerns	Helps map functional niches linked to progression and therapy resistance	[63]
14	General/spatial transcriptomics datasets	Graph neural networks	Spatial transcriptomics with location information	Spatial domain heterogeneity	Proposes a graph-deep-learning framework to improve spatial clustering across heterogeneous spatial transcriptomics datasets	Choice of graph module and scalability still matter	Relevant for discovering spatially distinct tumor regions	[64]
15	Pan-cancer	Cross-attention transformer multimodal fusion	Histology + genomics	Survival heterogeneity across cancer types	Uses multimodal fusion of pathology and genomic data for pan-cancer survival prediction, aiming to capture complementary phenotype–genotype signals	Multimodal alignment and external validation are challenging	Important for prognosis modeling in precision oncology	[65]

### 3.2. AI-Guided Biomarker Discovery and Multi-Omics Data Integration

Cancer diagnostic, prognostic, and therapeutic decisions require biomarkers. These biomarkers include genetic mutations, gene expression, proteins, and metabolic indicators. High-dimensional biological data is difficult to analyze. AI helps by using ML and DL methods to identify patterns associated with clinical outcomes [38,66,67]. AI is also better at combining multiple data types, such as genomics, transcriptomics, proteomics, and metabolomics. This approach provides a broad view of tumor biology by showing how genetic changes affect other molecular layers [68,69,70]. Some AI algorithms join TCGA genomic mutation data with gene expression and proteomic data. This enables researchers to simulate tumor signaling networks and identify key pathways or drug targets that TCGA alone may miss [69,71,72]. AI can also match multi-omics data with single-cell data to reconstruct tumor evolution and explain treatment resistance. This gives valuable knowledge for precision oncology [71,73].

**Figure 4:**
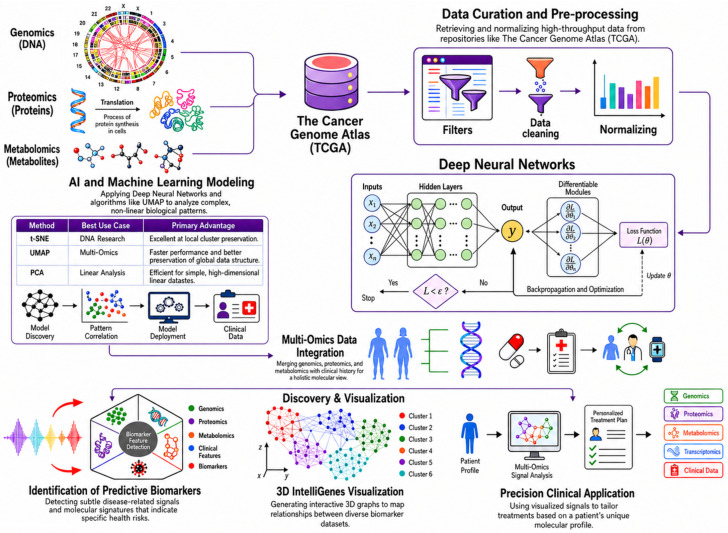
AI-Driven Multi-Omics Data Integration Pipeline for Cancer Biomarker Discovery, Network Visualization, and Precision Clinical Decision Support.

Figure 4 shows the AI-based workflow for biomarker discovery that uses multi-omics data. The process starts with collecting many types of biological data. These include genetics (DNA mutations), proteomics (protein expression), and metabolomics (metabolic profiles). Data is collected from large databases, such as TCGA. After collection, the data is checked and prepared by filtering, normalizing, and cleaning it. This keeps the data uniform and high-quality. After this step, AI and ML models such as DNNs, t-SNE, UMAP, and PCA are used. These tools find complex nonlinear trends and links in the combined datasets. These features, along with clinical data, let researchers fully integrate multi-omics data. This provides a complete view of tumor biology. This approach supports the identification of biomarkers and subtle patterns linked to cancer progression and patient outcomes. The workflow also uses advanced tools, such as 3D IntelliGenes network mapping. These tools help researchers study biomarker relationships across different biological layers. These insights may help create more precise clinical strategies by finding patient-specific molecular signatures [38,70,74].

## 4. AI-Guided Cancer Mechanistic Target Discovery and Translational Therapeutic Development

AI is increasingly being explored as a means of moving beyond descriptive molecular profiling toward the prioritization of functionally relevant genes, dysregulated pathways, and context-dependent cellular dependencies. Specific focus is put on how AI can be used to prioritize driver genes, dysregulated pathways, synthetic lethal interactions, and druggable molecular circuits, and how the results can be used to design therapeutic interventions based on structurally-aware modeling, virtual screening, drug repurposing, and rational combination design. Meanwhile, many existing AI and DL models are mostly data-driven predictive models, as opposed to completely mechanistic system biology models. Nevertheless, when thoughtfully combined with biological understanding and experimental validation, the methods offer a narrow and more useful model of connecting mechanistic cancer biology with AI-enabled therapeutic novelty.

### 4.1. AI for Functional Target Prioritization in Cancer

The ability to identify molecular-level changes and accurately classify them as treatable biological determinants is a significant challenge in oncology. Genomic, transcriptomic, epigenetic, and proteomic abnormalities are usually found in large numbers in tumors, with most of them being passenger events of limited functional importance. Analytical frameworks based on AI can help solve this issue by ranking candidate targets based on their functional relevance within molecular networks and disease-associated regulatory programs in molecular networks and disease-related regulatory programs [2]. Unlike traditional methods, which usually use individual biomarkers or manually crafted hypotheses, AI models can also analyze the complex nonlinear interactions between genes, proteins, signaling pathways, and microenvironmental factors to identify drivers of oncogenesis and tumor progression. Notably, in these models, statistical and systems-level associations are usually inferred using large-scale data, as opposed to modeling biological processes explicitly using a set of known physical or biochemical rules.

Such AI approaches that are based on networks are especially useful in defining key genes, bottleneck regulators, and disrupted pathway modules that are required to maintain tumors by modeling gene regulatory networks, protein-protein interaction networks, signaling cascades, and metabolic circuits. These types of systems-level analyses facilitate the identification of molecular dependencies that are likely not to be seen through single-gene analyses per se [75]. Besides, graph-based learning frameworks have the capability to reflect the relational architecture of biological systems and enhance the prioritization of targets based on network centrality, context-specificity, and inferred functional relevance. This descriptive to functional/systems-level target prioritization change is why AI would be particularly valuable in identifying robust therapeutic targets in complicated cancers [76]. In cases where a stronger causal or mechanistic interpretation is desired, this typically needs to be combined with causal modeling, perturbation data, or biologically constrained systems biology models.

### 4.2. Causal AI and Functional Inference in Mechanistic Target Discovery

Although many AI models are effective at identifying correlations, mechanistic cancer research increasingly requires approaches that can infer causality rather than association alone, as described in Table 3. AI has become an important emerging direction. Causal modeling frameworks, including directed acyclic graphs, counterfactual reasoning, and interventional inference strategies, can help distinguish molecular events that actively drive tumor progression from those that are merely correlated with disease status. This distinction is critical for target discovery because therapeutic development requires intervention on biologically meaningful drivers rather than statistically associated features [75].

Causal AI is particularly relevant in oncology, where multiple interacting pathways and feedback loops complicate the interpretation of observational omics data [76]. By integrating prior biological knowledge with data-driven inference, these methods can improve the identification of upstream regulators, causal signaling nodes, and tumor-specific dependencies [77]. This not only strengthens confidence in target prioritization but also enhances the biological interpretability of AI-generated predictions. As a result, causal AI represents a valuable step toward more mechanistically grounded and clinically translatable target-discovery pipelines.

### 4.3. AI-Enabled Discovery of Synthetic Lethal Vulnerabilities

Another major application of AI in mechanistic oncology is the discovery of synthetic lethal interactions. Synthetic lethality occurs when the simultaneous mutation of two genes results in the death of a cell, but the mutation of either of the genes is feasible. This principle has been of great significance in precision oncology since tumor cells usually bear prior genomic mutations, which provide selective vulnerability. With AI models, it is possible to analyze large-scale genomic, functional screening, and dependency data to identify gene pairs or pathways that display synthetic lethality to find high-quality therapeutic options [78]. The strength of this approach is that it takes advantage of tumor-related liabilities and reduces the toxicity of normal tissues. Models of ML and DL can combine mutation patterns, pathway activity, gene essentiality, and drug-response with the aim of identifying potential synthetic lethal partners in relation to clinically relevant oncogenic changes. AI can assist in a more rational design of specific therapies by connecting molecular malfunctions with exploitable weaknesses. In comparison to conventional screening methods, AI-assisted synthetic lethality discovery is more scalable and could be used to expedite the identification of new candidates to be subjected to experimental validation.

### 4.4. AI in Drug Target Validation, Repurposing, and Combination Therapy

In addition to candidate target identification, AI is also involved in other translational phases of therapeutic development, such as target validation, drug repurposing, and combination therapy design. After prioritization of a target, AI-based predictive models are capable of estimating drug-target binding, molecular affinity, structural compatibility, pathway perturbation, and toxicity risk. These computational techniques significantly reduce the search space to be experimentally tested and enhance the effectiveness of drug discovery at an early stage [79]. Drug repurposing is another area in which AI is being applied, particularly by matching existing or previously characterized compounds to newly prioritized cancer targets, pathway dependencies, or resistance-associated mechanisms using molecular signatures, chemical structure, pathway interactions, and biological network context. AI may support combination-therapy design by prioritizing drug pairs predicted to have complementary pathway effects or to overcome resistance-associated dependencies between drugs and forecasting which paired interventions are most apt to overcome resistance mechanisms or address multiple oncogenic dependencies at once [80]. These features are especially valuable in cancer, where single-agent therapies have a low probability of long-term success due to tumor evolution and pathway redundancy. Although AI-based models may assist in prioritizing candidate combination strategies, most reported findings remain computational or preclinical, and only limited evidence currently supports routine clinical translation of AI-derived combination regimens.

**Table 3:** Key Concepts and Applications of AI-Driven Mechanistic Cancer Target Discovery.

Concept	Description	Research Significance	Key Applications/Findings	Citations
**Network Biology & Systems Biology**	AI models analyze gene regulatory and protein–protein interaction networks to understand the architecture of cellular systems involved in cancer.	Enables identification of central regulatory nodes and molecular hubs that drive tumor progression.	Discovery of novel anticancer targets and improved understanding of tumor signaling networks.	[2,75]
**AI-Based Pathway Analysis**	ML integrates multi-omics datasets to detect dysregulated signaling pathways in cancer.	Provides systems-level understanding of cancer biology and helps identify molecular pathways suitable for targeted therapy.	Identification of oncogenic regulators and synergistic therapeutic targets in colon and gastric cancers.	[76]
**Causal AI & Mechanistic Modeling**	Use of DAGs, counterfactual reasoning, and causal inference models to identify cause-and-effect relationships in biological networks.	Distinguishes driver mutations from passenger mutations and improves target prioritization.	Identification of causal regulators of tumor progression and key driver genes.	[75]
**Synthetic Lethality Discovery**	AI predicts gene pairs whose combined disruption selectively kills cancer cells.	Enables the development of highly selective targeted therapies with minimal toxicity to healthy cells.	Discovery of synthetic lethal gene interactions for precision oncology strategies.	[77]
**AI-Driven Drug Target Identification**	DL models analyze molecular structures and biological interactions to predict drug-target binding.	Accelerates early-stage drug discovery and improves candidate selection.	Discovery of STK33 inhibitors that induce apoptosis and inhibit tumor cell proliferation.	[11]
**Multi-Target Drug Discovery**	AI models analyze biomarker signatures to design therapies that simultaneously target multiple molecular pathways.	Supports personalized medicine and improved treatment efficacy.	Improved drug response prediction and therapeutic design in colon cancer.	[80]
**AI-Assisted Drug Combination Screening**	Computational models simulate drug interactions within biological networks to identify synergistic combinations.	Helps overcome drug resistance and improve treatment outcomes.	Identification of synergistic targets for PKMYT1 inhibitors in metastatic gastric cancer models.	[76]
**AI in Drug development Pipeline**	AI assists in target identification, virtual screening, toxicity prediction, biomarker discovery, and clinical trial optimization.	Accelerates drug development while reducing cost and experimental workload.	Can support patient stratification, precision oncology strategies, and faster therapeutic development.	[6]

### 4.5. Translational Perspective and Remaining Challenges

AI-based mechanistic target discovery represents a shift from descriptive to actionable cancer data analysis. AI can also be used to identify biologically relevant and clinically tractable cancer targets in a more systematic manner by combining network modeling, causal inference, synthetic lethality prediction, and pharmacological modeling. Such a framework may help accelerate the development of precision therapies, improve patient stratification, and support more rational treatment design. Nevertheless, there are still several issues. The effectiveness of mechanistic AI solutions is highly contingent on the quality, completeness, and biological consistency of underlying data. Model architectures, data sources, and disease situations can also make varying predictions of the target, which points to the necessity of reproducibility, external validation, and experimental confirmation. Furthermore, translation of AI-prioritized targets to clinically useful therapeutics should be tightly coupled with computational modeling, functional biology, pharmacology, and clinical oncology. AI is increasingly being considered a facilitating technology for supporting the discovery of mechanistically informed, biologically grounded, and clinically relevant cancer therapies, as well as mechanistically inspired, biologically based, and clinically exploitable cancer therapies.

## 5. AI-Enabled Translation of Cancer Target Discovery into Therapeutic Development

In cancer drug discovery, AI plays an essential role when it is applied as a translational extension of the mechanistic target identification, as mentioned in Table 4. After prioritizing biologically relevant targets, pathway dependencies, or synthetic lethality vulnerabilities, AI-based frameworks can aid the subsequent steps in therapeutic development, which include drug-target interaction modeling, structure-informed screening, hit discovery, lead optimization, and mechanism-guided drug repurposing. In this context, AI is considered not as a general pharmaceutical platform, but as a translational framework for converting mechanistically prioritized cancer targets into testable therapeutic candidates, but in transforming mechanistically prioritized cancer targets into testable therapeutic candidates. In this way, the focus of this section is on the connection between AI and target discovery, which connects with the selection of compounds, molecular design, and early translational development [81,82,83,84].

### 5.1. From Target Prioritization to Hit Discovery and Lead Optimization

Once cancer targets have been prioritized by means of multi-omics integration, network modeling, or causal and functional inference, AI can be used to translate them into therapeutic candidates. A significant use is in structure-informed target characterization, where AI programs like protein structure prediction and drug-target interaction modeling are used to define ligandable pockets, functional domains, and molecular interaction constraints applicable in inhibitor design. Specifically, prediction of protein structure, including AlphaFold, has increased the ability to analyze the target proteins in a therapeutically relevant manner and to enable early-stage structure-guided drug discovery [84,85]. Such learnings can then be applied to virtual screening processes where AI-directed models will screen through large chemical libraries to find compounds with possible affinity to specific cancer targets.

Quantitative structure-activity relationship (QSAR) modeling, molecular docking, graph neural networks, and DL-based binding prediction are approaches that can speed up the process of hit discovery but can reduce the load of experimental screening [86,87,88]. After identifying hits, AI can also optimize leads by forecasting molecular characteristics in the development of therapeutics, such as binding affinity, selectivity, solubility, toxicity, and pharmacokinetics. These approaches can assist in giving a ranking of the compounds in the order of synthesis and biological testing, enhancing the efficiency of the early translational drug development phase. Most significant in the context of this review is their direct correlation with targets that have already been determined to be functionally relevant in cancer biology. By bridging this gap, AI can act as a viable intermediary between mechanistic target identification and the creation of experimentally actionable therapeutic targets [83,84].

### 5.2. AI-Guided Drug Repurposing and Rational Therapeutic Design

In addition to de novo screening and lead optimization, therapeutic development may also be facilitated by AI in the form of target-informed drug repurposing and rational molecular design. In cases where a target, pathway dependency, or resistance-related mechanism is already known, biological networks, chemical structures, pharmacological profiles, and multi-omics data can be combined in AI models to determine existing compounds with potential activity against the prioritized vulnerability [81,83,84]. The approach is particularly appealing in oncology, since repurposed drugs may already have partially characterized safety and pharmacokinetic profiles, which can reduce development timelines and lower costs in early-stage development [89]. Another application of AI to help with rational therapeutic design is the use of generative modeling approaches that generate or optimize novel molecules based on constraints related to targets of interest. The compounds with the targeted pharmacological and physicochemical characteristics can be designed using generative adversarial networks (GANs), variational autoencoders (VAEs), reinforcement learning, and similar deep learning systems [90]. AI can be employed not only for general molecular discovery but also for the design and optimization of candidate therapeutics directed toward biologically prioritized cancer targets. This translational application of generative AI can be observed in platforms such as Chemistry42 and related systems [90]. In that regard, the translational usefulness of AI in this section is that it allows the mechanistically based target hypotheses to be transformed into repurposed or novel therapeutic candidates that could be experimentally validated. Accordingly, AI-guided repurposing and rational therapeutic design should currently be viewed primarily as hypothesis-generating and prioritization frameworks that still require rigorous pharmacological, experimental, and clinical validation.

### 5.3. Scope and Translational Perspective

The application of AI during clinical trial optimization and other, later stages in pharmaceutical development is not the emphasis of this review. This article focuses on early translational research steps that directly lead to mechanistic target discovery, i.e., target-linked screening, compound prioritization, molecular optimization, and rational therapeutic design. Even in this more limited context, there are still critical issues, such as data quality, transparency of the algorithmic process, reproducibility, validation problems, and regulatory concerns. Solutions to the aforementioned concerns are necessary to make AI-directed therapeutic development biologically plausible, experimentally testable, and clinically translatable [91,92,93,94].

**Table 4:** AI applications in drug discovery and development, including key methods, representative AI platforms, benefits, challenges, and supporting references across different stages of the pharmaceutical research pipeline.

Stage of Drug Development	AI Application	Description of AI Role	Key Methods/Technologies	Representative AI Tools/Platforms	Major Benefits	Key Challenges	Citations
Target Discovery	Drug Target Identification	AI analyzes genomic, proteomic, and multi-omics datasets to identify disease-associated genes, proteins, and biological pathways as potential drug targets. AI also predicts protein structures and drug–target interactions.	Multi-omics data integration, ML prediction models, protein structure prediction, network biology, drug–target interaction modeling	AlphaFold 3 v3.0.2, DeepMind AlphaFold DB, DeepChem 2.8.0, Open Targets Platform 26.03, BioBERT v1.1, DrugBank API v1 AI tools	Faster identification of therapeutic targets; improved understanding of disease mechanisms; higher success rate in early-stage discovery	Data quality issues, biased biological datasets, validation difficulties, and model interpretability limitations	[84,85,93]
Hit Discovery	Virtual Screening	AI enables screening of millions of chemical compounds against biological targets to identify potential drug candidates. Computational models predict molecular binding affinity and biological activity.	Molecular docking, QSAR modeling, DL prediction models, graph neural networks, and high-throughput virtual screening	AtomNet, DeepChem (2.6.0), Schrödinger AI Platform 2026-1 , AutoDock Vina v1.2.6, MOE (Molecular Operating Environment)	Efficient identification of promising drug candidates; reduced experimental cost; accelerated hit discovery	High computational cost; complex molecular representations; limited interpretability	[82,95]
Lead Optimization	AI-Driven Drug Design	AI predicts physicochemical and pharmacokinetic properties such as toxicity, solubility, and binding affinity, enabling optimization of candidate molecules before synthesis.	DL models, molecular property prediction, QSAR models, molecular dynamics simulations, graph neural networks	DeepChem, Chemprop 2.2.3, Schrödinger AI Drug Design Suite, IBM RXN for Chemistry	Faster optimization of lead compounds; reduced experimental trials; improved molecular design	Model explainability limitations; difficulty predicting complex biological interactions	[96,97,98]
Drug Development	Drug Repurposing	AI identifies new therapeutic uses for existing drugs by integrating chemical, biological, genomic, and clinical data. This approach is widely used for emerging diseases and rare conditions.	Network-based analysis, ML prediction models, knowledge graph analysis, and multi-omics integration	DrugRepAI, DeepPurpose 0.1.5, RepurposeDB, BenevolentAI Platform	Lower development cost; shorter regulatory pathways; faster therapeutic availability	Data heterogeneity, experimental validation requirements, and regulatory challenges	[81,83,84]
Molecular Innovation	Generative AI in Drug Discovery	Generative AI creates novel chemical structures and optimizes molecules with desired pharmacological properties using advanced DL models.	Generative adversarial networks (GANs), variational autoencoders (VAEs), transformer models, reinforcement learning	Insilico Medicine (Chemistry42), DeepGenChem, REINVENT 4.7, MolGAN, Generative Tensorial Reinforcement Learning (GENTRL 0.1)	Enables rapid generation of candidate molecules; accelerated lead discovery; optimization of drug properties	Synthetic feasibility concerns, unrealistic molecule generation, and model interpretability challenges	[82,90,99]
Clinical Development	AI-Based Clinical Trial Optimization	AI enhances clinical trial design, patient recruitment, eligibility matching, and outcome prediction using large healthcare datasets and real-world evidence.	Electronic health record analysis, predictive analytics, digital twins, synthetic control arms, ML outcome prediction	Tempus AI, TriNetX, IBM Watson Health, Deep 6 AI, Medidata AI Clinical Platform	Reduced trial duration and cost; improved patient stratification; increased trial success rates	Ethical concerns, patient privacy issues, regulatory barriers, heterogeneous clinical data integration	[91,92,100]
Entire Drug Discovery Pipeline	AI-Driven Drug Discovery Ecosystem	AI integrates across the entire pharmaceutical R&D pipeline, from early discovery to clinical development, enabling data-driven decision-making and accelerating therapeutic innovation.	ML, DL, big data analytics, bioinformatics, cheminformatics, knowledge graphs	DeepChem, Insilico Medicine, BenevolentAI, Exscientia, and Recursion Pharmaceuticals Platform	Reduced development timelines and costs; improved precision medicine; enhanced predictive capabilities	Data bias, lack of standardization, reproducibility issues, and regulatory uncertainty	[93,94,101]

## 6. Case Studies Linking AI-Based Target Discovery to Therapeutic Development of Cancer

Instead of providing general case studies of AI in all fields of precision oncology, this section dwells specifically on examples where AI has been applied to find or rank a cancer target, describe its therapeutic utility, and direct the identification or optimization of candidate compounds, as mentioned in Table 5. These examples are particularly critical since they show that the multi-omics integration, network-based analysis, and generative design may go beyond descriptive cancer biology and directly impact therapeutic approaches [2,6,75,102].

### 6.1. AI-Guided Target Prioritization and Small-Molecule Discovery

A representative example of AI-guided target prioritization and small molecule discovery is the development of CDK20 inhibitors for hepatocellular carcinoma using an integrated AI workflow. AlphaFold-predicted protein structures were used with PandaOmics to identify targets and Chemistry42 to design novel molecules. CDK20 was identified as a high-priority AI-supported therapeutic target, and the generative platform produced 8918 candidate molecules. Based on the initial round of the experiments, seven compounds were synthesized, one of which was ISM042-2-001, which was subsequently refined to ISM042-2-048 with nanomolar potency [75,102,105]. This example is particularly valuable because it illustrates a complete translational pathway: AI-based target prioritization, structure-based molecular design, synthesis, and potency optimization.

Another interesting application is the discovery of ENPP1 inhibitor ISM5939 for treating solid tumors. AI-assisted multi-omics target ranking and generative chemistry were employed in this study, leading to the identification of ENPP1 as a therapeutically relevant tumor immune-checkpoint target. The resulting orally active inhibitor increased STING pathway signaling and was synergistic with PD-1/PD-L1-directed immunotherapy [6,12,106]. The translational value of AI in genomics demonstrates how computational target identification can be linked to mechanism-based therapeutic design and biologically relevant anticancer activity, as it demonstrates how computational identification of the target can be directly connected to the mechanism-based design of therapeutics and biologically relevant anticancer activity [107].

### 6.2. AI for Multi-Target and Pathway-Oriented Therapeutic Prioritization

AI can also be used to aid in developing therapeutic methods where cancer treatment involves the concomitant consideration of a number of genes, pathways, or resistance-associated mechanisms. For example, drug target prioritization and candidate selection in colon cancer have been performed with machine learning methods that combine gene expression, mutation profile, and protein interaction networks, which have shown high predictive accuracy in identifying therapeutically relevant targets and drug-response relationships [103]. Likewise, deep learning systems like PASO that are pathway-aware combine multi-omics pathway information with drug chemical structures to facilitate biologically informed predictions of anticancer drug response [12,108].

This review focuses on the ability of these models to bridge biological dependencies at the pathway level with the logic of therapeutic design. Such studies are pertinent because cancer is usually driven by interplaying pathway networks and resistance programs rather than a single molecular change. AI models that use the context of pathways can thus be used to refine therapy, select combinatorial intervention, and more appropriately align drug development to the mechanistic characteristics of tumor biology [12,76,80].

### 6.3. Translational Significance and Remaining Challenges

Combined, these case studies show that the most useful uses of AI in cancer therapeutics are those that combine target discovery with generating compounds, optimization, or mechanism-directed therapeutic selection. They demonstrate that AI can be used not only to identify the vulnerabilities of cancer but to transform these vulnerabilities into candidate strategies of treatment in practice [2,6,75,102]. However, challenges regarding data quality, model interpretability, reproducibility, and the requirement of strong biological and experimental validation before clinical translation continue to exist [40]. In the future, increased collaboration among AI, mechanistic cancer biology, medicinal chemistry, and experimental oncology will be required to better translate computational predictions into effective anticancer therapies. Although these case studies demonstrate meaningful translational progress, they should be interpreted primarily as examples of promising preclinical or early translational advancement rather than as evidence that AI-enabled therapeutic pipelines are already broadly validated in routine clinical oncology.

## 7. Emerging Directions in AI for Mechanistic and Translational Oncology

**Figure 5:**
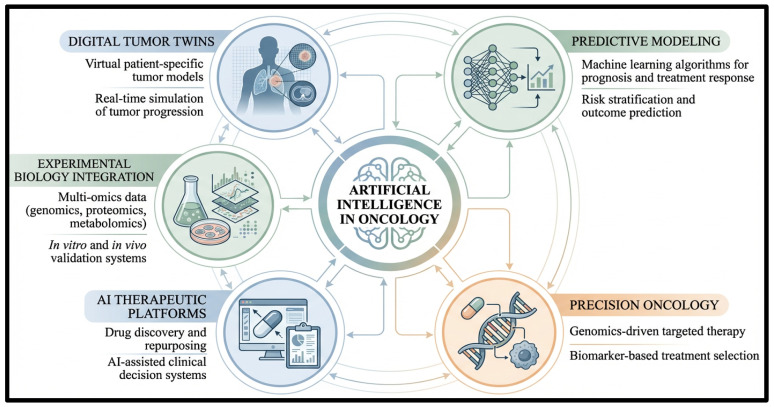
Convergence of AI with Emerging Frontiers in Oncology. Integration of digital tumor twins, predictive modeling, precision oncology, AI therapeutic platforms, and experimental biology for next-generation cancer research.

In its role not as a general clinical support technology, but as a mechanistically informed system of integrating tumor biology with therapeutic development, AI is likely to have the most significant impact in the field of oncology. In this sense, the most topical emerging directions are those that will enhance the determination of actionable cancer vulnerabilities, enhance the biological explanation of tumor dependencies, and hasten the transfer of such results into testable treatment approaches [109,110]. Figure 5 demonstrates the combination of predictive modeling, digital tumor twins, experimental biology, and data integration frameworks to give a conceptual picture of how future AI systems can be used to support a more mechanistically based oncology pipeline. This review considers these future directions mainly in the context of their usefulness in target discovery, target validation, resistance modeling, and therapeutic design, as opposed to being general precision oncology platforms.

### 7.1. Digital Tumor Twins for Mechanism-Based Therapeutic Testing

The creation of digital tumor twins, which are computational models designed to replicate patient-specific tumor biology and simulate disease progression and therapeutic response, represents one of the most promising future directions [111,112]. Digital tumor twins are especially topical to this review, as they can be used as translational instruments to bridge the gap between mechanistic target knowledge and in silico therapeutic testing [113,114].

Instead of being mentioned here as generic personalized medicine platforms, digital tumor twins are best applicable when they are applied to assess the effects of targeting particular molecular vulnerabilities, predict resistance mechanisms, and aid in the development of rational combination therapies. The use of AI-based tumor modeling systems, which include TumorScope, is an example of how spatial and molecular tumor data could be combined to predict drug distribution, tumor metabolism, and probable treatment response [115,116]. By so doing, digital tumor twins can contribute to extending AI beyond target prioritization to functionally informed treatment simulation. Nonetheless, they will still require high-quality longitudinal data, multimodal integration, and the formulation of interpretable models that can be validated both biologically and clinically [111,112,113,114,115,116,117].

### 7.2. Integration of AI with Experimental Biology for Target Validation

**Figure 6:**
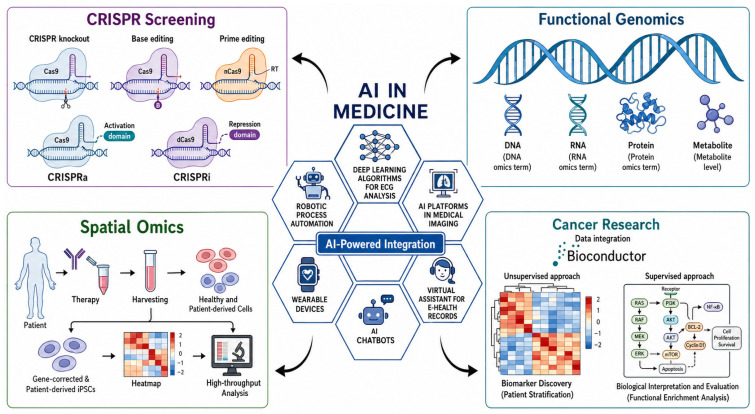
Conceptual framework illustrating the integration of AI with experimental biology platforms, including CRISPR screening, functional genomics, and spatial omics, to enable data integration, biomarker discovery, and patient stratification in cancer research.

The further close association of AI with experimental biology platforms capable of supporting mechanistic hypotheses is the next direction with an extremely high priority. Figure 6 depicts how AI can be combined with CRISPR screening, functional genomics, and spatial omics to create biologically informed data on target discovery and therapeutic prioritization [118,119]. This overlap is particularly noteworthy considering that many of the existing AI systems are predictive, as opposed to experimentally validated, which is one of the key limitations of the existing systems.

CRISPR-based perturbation technologies, such as knockout, activation, interference, base editing, and prime editing techniques, can provide powerful platforms to test gene function in cancer [120,121]. With AI, these data sets can be utilized to infer dependencies between genes, derive networks of interactions between genes and their functions, and rank targets associated with tumor growth, metastasis, immune evasion, and drug resistance [122,123]. AI can also combine functional genomics and multi-omics data to determine regulatory patterns, pathway dependencies, and potential biomarkers of cancer progression and response to treatment [124,125]. Spatial omics is an extra mechanistic dimension as it maintains tissue architecture and can be used to study tumor–microenvironment interactions, cellular organization, and immune context [126]. Collectively, these strategies enhance a shift towards descriptive analysis of molecules to experimentally justified target discovery.

### 7.3. Future Mechanistic AI Frameworks for Target Discovery and Therapeutic Design

Another major direction in the future is the creation of AI systems that integrate multimodal with causal reasoning, pathway modeling, and functional dependency analysis. This direction is supported by the experimental framework summarized in Figure 5. Practically, this implies taking genomic modifications, gene-dependency information, pathway activity, and spatial or microenvironmental setting into models that can produce mechanistically interpretable therapeutic hypotheses.

The latter methods are particularly useful when it comes to finding druggable cancer circuits, rewiring linked to resistance, and ranking rational combination strategies. Indicatively, the genomic dependency screens and pathway data analyses with the assistance of AI have already expedited the discovery of synthetic lethal interactions and related therapeutic opportunities, such as the BRCA1/BRCA2-associated vulnerabilities [127,128,129]. In the future, AI systems integrating computational prediction and perturbation data with biological knowledge and experimental validation are likely to become indispensable in the field of translational oncology. This will be essential to ensure that future AI-directed oncology studies are mechanistically plausible and therapeutically actionable.

### 7.4. Public Databases Supporting AI-Driven Cancer Research and Drug Discovery

**Figure 7:**
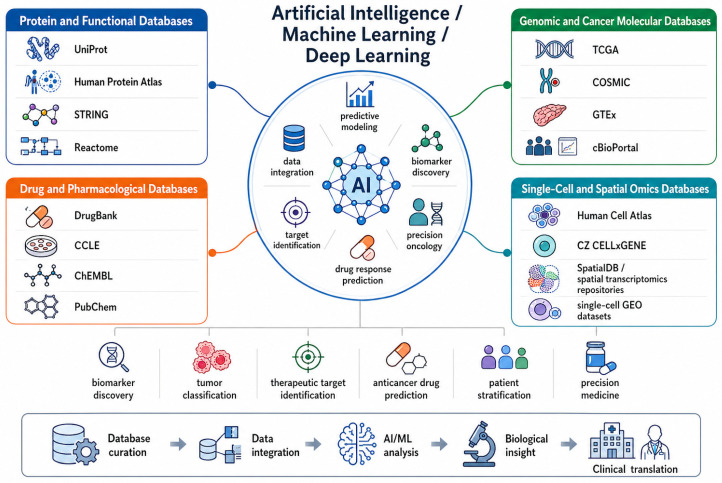
AI-Driven Integration of Biomedical Databases for Drug Discovery and Precision Medicine.

Access to large, public, well-annotated biomedical databases is necessary for the success of AI and ML applications in cancer research, as it enables the training, validation, and accurate interpretation of models using molecular and clinical data. Figure 7 illustrates the key resources that constitute this data ecosystem, including UniProt for protein sequence and functional annotation, TCGA and GTEx for cancer and normal tissue genomic and transcriptomic profiles, COSMIC for somatic mutation information, DrugBank for drug and target knowledge, and CCLE for cancer cell line characterization and drug response. Moreover, recent data sources like the Human Cell Atlas and CZ CELLxGENE can be considered useful data of single cells that facilitate further analyses regarding tumor heterogeneity and the tumor microenvironment. Collectively, these databases are used as the initial knowledge base to generate AI-based biomarker discovery, tumor classification, therapeutic target identification, anticancer drug prediction, and precision oncology solutions.

## 8. Challenges and Limitations of AI in Cancer Research

AI has advanced cancer research by improving diagnosis, treatment planning, and drug discovery. However, data, model, ethical, and regulatory concerns limit the effective use of AI in oncology. Addressing these challenges is essential to ensure AI is safe, reliable, and equitable in cancer care.

### 8.1. Technical and Methodological Challenges

Data quality and bias are two major problems in AI-based cancer research. AI systems require large datasets for training. Most accessible medical datasets are incomplete or disparate they are collected using different methods or formats. These datasets often do not represent diverse populations. Many are biased toward certain geographic areas or ethnicities. This leads to biased predictions and unequal healthcare outcomes. As a result, AI models can work effectively with some patient groups but fail for underrepresented ones. This worsens health disparities in cancer care [130,131]. Dataset imbalance can also arise from unequal representation of age, sex, and disease stages (such as early vs. advanced cancer). It can also come from cancer subtypes (different types of cancer within a tissue), imaging platforms (various technologies used to scan bodies or tissues), and treatment histories. All of these factors can influence model behavior. Missing data, inconsistent annotation standards (differences in how data is labeled), and variable Sample collection or sequencing (inconsistencies in how biological samples are collected or analyzed) can further reduce reliability.

The interpretability of AI models, especially deep learning (DL) systems, remains a notable limitation. Many advanced algorithms operate as black-box models with internal workings invisible to users, generating predictions without clear explanations. This lack of transparency makes AI-generated recommendations hard for clinicians to comprehend, verify, and trust. In clinical settings where technology affects patient survival, unclear AI outputs are a barrier [132,133,134]. This is especially important in oncology, where decisions involve high-risk treatment selection, prognosis (the likely disease outcome), and prediction of therapy response (how a patient may respond to treatment). Without interpretability or meaningful explanations, AI adoption may remain limited, even if predictive accuracy is high.

There are reproducibility issues in AI research in oncology. Many studies lack enough methodological detail on data preprocessing, model structure, or training. As a result, other researchers may not be able to reproduce results or validate models elsewhere. AI models trained at a single institution should not be generalized to other hospitals or populations [131]. Generalizability is closely related to reproducibility. A model built with data from one institution or group may work well internally but fail on independent datasets. Differences in demographics, disease prevalence, laboratory methods, imaging equipment, workflows, and data pipelines can greatly affect performance. External validation across diverse populations is required. AI models must be robust and transferable for broad clinical use.

AI technologies face limited applications due to high computational demands. Developing advanced ML models requires high-performance hardware such as graphical processing units (GPUs), ample digital storage, and extensive computing resources. Many healthcare organizations struggle to meet these requirements, not just in low- and middle-income countries but globally. Access to AI-based cancer research is often restricted [135]. Multimodal and foundation models that use multiple data sources such as imaging (X-rays or MRIs), genomic (DNA), transcriptomic (gene expression), proteomic (protein levels), and clinical (patient records) data increase the burden. These models require substantial memory, long training times, specialized infrastructure, and ongoing maintenance, hindering use in resource-constrained clinical settings.

Another important limitation in AI-assisted cancer drug discovery is the high attrition rate of computationally prioritized candidates during preclinical and downstream development. Although AI can accelerate target identification, virtual screening, and lead optimization, many candidates fail during experimental validation. They may not demonstrate sufficient biological efficacy, safety, pharmacokinetic stability, or acceptable toxicity. More broadly, the overall failure rate in drug development exceeds 90%. This underscores that computational prioritization alone is insufficient for successful therapeutic translation. Often, AI models are optimized for pattern recognition rather than for biological causality. This can lead to over-prioritization of compounds with limited real-world potential. To address this challenge more cost-effectively, AI-driven pipelines should be paired with early-stage experimental triage, multi-parameter ADMET filtering, and biologically relevant preclinical validation strategies. These steps help eliminate weak candidates before expensive downstream testing. This approach may improve resource allocation, reduce avoidable attrition, and increase the translational value of AI-guided oncology drug discovery.

### 8.2. Ethical and Regulatory Challenges

Besides technical constraints, AI in cancer research raises critical ethical questions. One such concern is patient data privacy. AI relies on large databases of medical data, including images, genetic data, and medical histories. Ensuring patient confidentiality and building trust in healthcare systems requires strong data governance and informed consent. This is even more complex in multi-institutional and international studies. Data sharing is essential but can be restricted by legal, institutional, and ethical considerations [136].

Another ethical concern is algorithmic bias, where AI models are trained on biased datasets. Without diverse and unbiased datasets, AI can produce discriminatory results that disadvantage certain patient groups. To address bias, datasets must be carefully designed. AI performance must be constantly monitored. Fairness-focused algorithms must be developed. Fairness assessment should become a core part of model evaluation, especially for AI tools used in clinical decision support [130,133].

Regulation is a key barrier to the implementation of clinical AI in cancer management. There is currently no single framework for evaluating or approving AI-based medical tools. Before using AI systems in patient care, regulatory bodies must ensure compliance with high safety, accuracy, and reliability standards. Legal responsibility for AI-assisted clinical decisions is still unclear. Safely implementing AI in oncology will require clear regulatory guidelines and international standards [132,136]. Continuously learning AI systems also creates regulatory challenges. Model behavior changes with new data. This raises questions about post-deployment monitoring, model updates, auditability, and long-term accountability.

AI has great potential to revolutionize cancer research and clinical oncology, but it also raises substantial technical, ethical, and regulatory issues. To overcome these constraints, an interdisciplinary approach, better data control, clearer AI systems, and robust regulatory frameworks will be needed to ensure AI technologies are used safely and fairly in cancer treatment. Future progress will depend not only on improved algorithms but also on standardized benchmarking practices, transparent reporting, and external validation across diverse populations. Stronger collaboration among computational scientists, clinicians, regulatory authorities, and biomedical researchers will be required. Such efforts are essential to improve the reproducibility, transferability, and equitable clinical deployment of AI models in oncology.

## 9. Future Perspectives of Artificial Intelligence in Cancer Research

AI will increasingly drive cancer research and clinical oncology. One key goal is to create hybrid AI models that combine large foundation models, which are AI systems trained on vast amounts of data and capable of performing multiple tasks, with causal AI methods, which focus on identifying cause-and-effect relationships. Classical AI systems spot trends in big data but can’t say why those trends exist. Adding causal reasoning helps explain the “why” behind genetic mutations (changes in DNA), environmental factors (outside influences affecting health), and tumor development (how tumors grow and change). This enables researchers and clinicians to make more reliable, science-based decisions by improving model interpretability, meaning the AI’s reasoning is easier to understand. Another advance is combining data types, including genomics (the study of genes and DNA), proteomics (the study of proteins), radiomics (the analysis of imaging data such as CT or MRI scans), and clinical records (patient health information). Because cancer is complex and varied, integrating diverse data gives AI a clearer view of tumor biology. Advanced methods enable scientists to process complex datasets simultaneously, improving diagnosis, risk prediction, and the identification of disease subtypes.

AI will be vital in improving cancer treatment and clinical decision-making. The next generation of AI will rapidly process large patient-specific datasets, including molecular profiles, genetic mutations, imaging results, and treatment history, to identify the most effective individual therapies. This targeted approach will improve outcomes and reduce unnecessary side effects. Combined with clinical decision support systems, AI will help providers analyze complex data and make evidence-based decisions. These systems enable clinicians to detect cancer early, stage tumors, select treatments, and monitor patients by providing real-time insights from vast medical datasets. As these technologies advance, researchers, clinicians, and technology developers must collaborate to ensure that AI tools are reliable, transparent, and well-integrated into clinical practice. These developments will accelerate scientific discovery and enhance diagnosis, treatment, and care for cancer patients.

## 10. Conclusions

This review does not discuss AI as a general oncology tool but focuses on integrating diverse biological data: multi-omics (genomics, transcriptomics, proteomics, etc.), single-cell (from individual cells), spatial (cell and molecule locations in tissue), and functional (biological roles and activities of genes/proteins). These integrations reveal driver genes (genes whose changes promote cancer), dysregulated pathways (malfunctioning biological pathways), tumor dependencies (features cancer cells rely on), and actionable vulnerabilities (cancer weaknesses for treatment). This review demonstrates AI’s critical and growing role in elucidating cancer mechanisms and translating insights into drug development. AI goes beyond describing cancer profiles; it enables biologically informed interpretation of tumor biology and prioritization of drug targets. Additionally, AI advances therapeutic applications with network-based modeling (biomolecular interactions), causal inference (identifying cause-and-effect), structure-informed analysis (molecular shapes), virtual screening (in silico testing of potential drugs), generative design (computational creation of molecules), and drug repurposing (finding new uses for existing drugs). Despite ongoing challenges with data quality, interpretability, reproducibility, and empirical confirmation, the evidence here shows that AI increases the accuracy, efficiency, and impact of discovering new cancer drug targets. This review concludes that AI will be foundational and transformative in merging cancer biology insights to drive the next generation of more effective therapies.

## Figures and Tables

**Table 1:** Artificial Intelligence Technologies, Tools, Applications, and Future Perspectives in Cancer Research and Precision Oncology.

Methodological Level	AI Model/Platform Category	Core Methods/Models	Major Applications in Cancer Research	Representative Tools/Platforms	Key Benefits	Limitations/Challenges	References
Classical ML models	Tree-based, kernel-based, and statistical learning models	Random forests, support vector machines, gradient boosting, logistic regression, clustering	Cancer classification, biomarker discovery, survival prediction, treatment-response modeling	DeepVariant, GATK	Effective for structured clinical and omics data; often more interpretable than deep models	Performance depends on feature engineering and large annotated datasets; risk of bias.	[14,15,16,30]
Deep neural network models	Feedforward and sequence/image DL models	Artificial neural networks, convolutional neural networks (CNNs), and recurrent neural networks (RNNs)	Histopathology analysis, tumor detection, medical imaging, and mutation prediction	PathAI, NVIDIA Clara	Automated feature extraction and high predictive performance in complex data	Limited interpretability; high computational demand; dependence on large labeled datasets	[20,21,32]
Transformer and foundation models	Large pretrained biological and multimodal models	Transformers, self-supervised learning, transfer learning, large language/biological foundation models	Protein structure prediction, molecular design, drug discovery, and biomedical text mining	AlphaFold, NVIDIA BioNeMo, DeepChem	Captures complex long-range patterns and supports transfer across tasks	High computational cost, model complexity, and limited explainability	[14,24]
Graph-based AI models	Network and relational learning models	Graph neural networks, biomedical knowledge graphs, network-based inference	Drug–target interaction prediction, pathway analysis, target prioritization, systems oncology	Knowledge graph AI platforms	Reveals hidden biological relationships and supports network medicine approaches	Integration of heterogeneous data remains challenging	[30]
Multimodal integration models	Cross-modal data fusion models	Joint learning from imaging, genomic, transcriptomic, proteomic, and clinical data	Tumor characterization, prognosis prediction, patient stratification, precision oncology	Tempus AI	Enables comprehensive modeling of heterogeneous cancer data	Requires harmonized, large-scale integrated datasets	[31]
Domain-specific imaging AI applications	Imaging and pathology AI is built mainly on DL.	CNN-based imaging analytics, radiomics pipelines, digital pathology models	Tumor grading, lesion detection, radiotherapy planning, and histopathology classification	DeepMind Radiotherapy AI, PathAI	Supports non-invasive assessment and improves diagnostic workflow	Imaging variability, standardization issues, and dataset annotation burden	[32,33,34]
Language-based AI applications	Clinical and biomedical natural language processing	Text mining, biomedical named-entity recognition, transformer-based language models	Mining literature, clinical notes, reports, and genomic annotations	IBM Watson for Oncology	Extracts insights from large unstructured biomedical text corpora	Data privacy concerns and difficulty handling noisy clinical text	[35,36]
Drug discovery AI platforms	AI systems for therapeutic discovery and repurposing	Virtual screening, molecular docking, generative modeling, predictive toxicity modeling	Anticancer drug discovery, drug repurposing, compound prioritization	DeepChem	May improve efficiency in early-stage drug discovery and improve drug–target prediction	Predictions require experimental and clinical validation	[24,30]
Clinical decision and translational AI platforms	AI-enabled clinical support systems	Predictive analytics integrating clinical, imaging, and genomic data	Personalized therapy selection, prognosis estimation, and treatment optimization	IBM Watson for Oncology, Tempus AI, OncoKB	Supports data-driven clinical decision-making in precision oncology	Limited real-world validation, interpretability, and regulatory concerns	[37,38,39]
Emerging patient-specific simulation platforms	Digital twin and trial-optimization AI	Computational patient modeling, predictive simulation, and patient stratification algorithms	Patient-specific treatment-response prediction, clinical trial design, and recruitment optimization	Digital twin oncology platforms, AI trial analytics platforms	Enables personalized simulation and may improve trial efficiency	High computational complexity, limited validation, and ethical/regulatory barriers	[40,41]

**Table 5:** Case Studies of AI Applications Across the Cancer Drug Discovery and Precision Oncology Pipeline.

Application Area	Case Study/AI Model	Description of AI Approach	Key Outcome/Example	Citations
AI-identified drug candidates	AlphaFold + PandaOmics + Chemistry42 (CDK20 inhibitor discovery)	AlphaFold-predicted protein structures were integrated with PandaOmics target identification and Chemistry42 generative AI to design small-molecule inhibitors.	Generated 8918 candidate molecules, synthesized 7 compounds, discovered ISM042-2-001, and optimized to ISM042-2-048 with nanomolar potency for hepatocellular carcinoma.	[75,102]
AI-identified drug candidates	Generative AI design of ENPP1 inhibitor (ISM5939)	Multi-omics target ranking and generative chemistry used to design immune-checkpoint inhibitors.	Developed oral ENPP1 inhibitor ISM5939, enhanced STING signaling, and showed synergy with PD-1/PD-L1 immunotherapy.	[6,12]
Multi-target drug discovery	ML + Adaptive Bacterial Foraging + CatBoost	Integrated gene expression, mutation profiles, and protein interaction networks for drug target prioritization.	Achieved 98.6% classification accuracy for colon cancer drug-response prediction and candidate selection.	[103]
Drug response prediction	ResGitDR (Interpretable DL)	Learns cancer cell state from somatic genome alterations and predicts drug sensitivity.	Improved prediction accuracy compared with traditional genomic models and works on both cell lines and patient data.	[2]
Drug response prediction	Deep Neural Networks for therapy response prediction	DNN models trained on pharmacogenomic datasets and clinical cohorts.	Predicted drug response and survival outcomes better than conventional ML approaches.	[12]
Drug response prediction	PASO pathway-aware DL	Integrates multi-omics pathway features with drug chemical structures.	Provides biologically meaningful predictions for anticancer drug response in precision oncology.	[12]
Precision oncology platform	CAN-Scan platform	ML applied to molecular testing and patient-derived cancer cells.	Identifies chemotherapy resistance mechanisms and suggests alternative therapies for colorectal cancer.	[40]
AI-guided biomarker discovery	Predictive Biomarker Modeling Framework (PBMF)	Contrastive learning applied to clinical datasets to identify predictive biomarkers.	Detects biomarkers associated with improved survival and immunotherapy response.	[104]
Biomarker discovery using digital pathology	DL on whole-slide pathology images	Computational pathology models extract prognostic biomarkers from histology slides.	Identifies image-derived biomarkers useful for patient stratification and treatment planning.	[103]
Tumor microenvironment prediction	DL on digital histopathology	AI predicts tumor microenvironment composition and immunotherapy response.	Enables identification of patients likely to benefit from immune checkpoint therapy.	[103]
Clinical biomarker detection	Pathology foundation models for lung cancer	Large-scale foundation models trained on histopathology images.	Demonstrated real-world biomarker detection for lung cancer precision oncology.	[40]

## Data Availability

No new data were created or analyzed in this study. Data sharing is not applicable to this article.

## References

[1] Di Mauro A., Berretta M., Santorsola M., Ferrara G., Picone C., Savarese G., Ottaiano A. (2025). Towards post-genomic oncology: Embracing cancer complexity via artificial intelligence, multi-targeted therapeutics, drug repurposing, and innovative study designs. Int. J. Mol. Sci..

[2] You Y., Lai X., Pan Y., Zheng H., Vera J., Liu S., Deng S., Zhang L. (2022). Artificial intelligence in cancer target identification and drug discovery. Signal Transduct. Target. Ther..

[3] Lu Y., Huang W., Li Y., Xu Y., Wei Q., Sha C., Guo P. (2025). Leveraging artificial intelligence in antibody-drug conjugate development: From target identification to clinical translation in oncology. npj Precis. Oncol..

[4] Liu H., Ma Y., Chen W., Gu X., Sun J., Li P. (2025). Strategies for the drug development of cancer therapeutics. Front. Pharmacol..

[5] Le M.H.N., Nguyen P.K., Nguyen T.P.T., Nguyen H.Q., Tam D.N.H., Huynh H.H., Huynh P.K., Le N.Q.K. (2025). An in-depth review of AI-powered advancements in cancer drug discovery. Biochim. Biophys. Acta (BBA)-Mol. Basis Dis..

[6] Sarvepalli S., Vadarevu S. (2025). Role of artificial intelligence in cancer drug discovery and development. Cancer Lett..

[7] Sufyan M., Shokat Z., Ashfaq U.A. (2023). Artificial intelligence in cancer diagnosis and therapy: Current status and future perspective. Comput. Biol. Med..

[8] Si H., Kumar S., Lata S., Ahmad A., Ghosh S., Stephansen K., Nagarkar D., Zhou E., Higgs B.W. (2025). Mechanistically explainable AI model for predicting synergistic cancer therapy combinations. Curr. Oncol..

[9] Messina P., Luciano B., Placente D. (2020). Tomorrow’s Healthcare by Nano-Sized Approaches: A Bold Future for Medicine.

[10] Sobhani N., Kugeratski F.G., Venturini S., Roudi R., Nguyen T., D’angelo A., Generali D. (2025). AI-based cancer models in oncology: From diagnosis to ADC drug prediction. Cancers.

[11] Tran N.L., Kim H., Shin C.-H., Ko E., Oh S.J. (2023). Artificial intelligence-driven new drug discovery targeting serine/threonine kinase 33 for cancer treatment. Cancer Cell Int..

[12] Albani F.G., Alghamdi S.S., Almutairi M.M., Alqahtani T. (2025). Artificial intelligence-driven innovations in oncology drug discovery: Transforming traditional pipelines and enhancing drug design. Drug Des. Dev. Ther..

[13] Molla G., Bitew M. (2024). Revolutionizing personalized medicine: Synergy with multi-omics data generation, main hurdles, and future perspectives. Biomedicines.

[14] Zhang B., Shi H., Wang H. (2023). Machine learning and AI in cancer prognosis, prediction, and treatment selection: A critical approach. J. Multidiscip. Healthc..

[15] Kourou K., Exarchos K.P., Papaloukas C., Sakaloglou P., Exarchos T., Fotiadis D.I. (2021). Applied machine learning in cancer research: A systematic review for patient diagnosis, classification and prognosis. Comput. Struct. Biotechnol. J..

[16] Tiwari A., Mishra S., Kuo T.-R. (2025). Current AI technologies in cancer diagnostics and treatment. Mol. Cancer.

[17] Fountzilas E., Pearce T., Baysal M.A., Chakraborty A., Tsimberidou A.M. (2025). Convergence of evolving artificial intelligence and machine learning techniques in precision oncology. Digit. Med..

[18] Kumar A., Metta D. (2024). AI-driven precision oncology: Predictive biomarker discovery and personalized treatment optimization using genomic data. Int. J. Adv. Res. Publ. Rev..

[19] Restrepo J.C., Dueñas D., Corredor Z., Liscano Y. (2023). Advances in genomic data and biomarkers: Revolutionizing NSCLC diagnosis and treatment. Cancers.

[20] Khalighi S., Reddy K., Midya A., Pandav K.B., Madabhushi A., Abedalthagafi M. (2024). Artificial intelligence in neuro-oncology: Advances and challenges in brain tumor diagnosis, prognosis, and precision treatment. Precis. Oncol..

[21] Shimizu H., Nakayama K.I. (2020). Artificial intelligence in oncology. Cancer Sci..

[22] Kumar V., Iqbal M.I., Rathore R. (2025). Natural Language Processing (NLP) in disease detection—A discussion of how NLP Techniques can be used to analyze and classify medical text data for disease diagnosis. AI in Disease Detection: Advancements and Applications.

[23] Sim J.-A., Huang X., Horan M.R., Baker J.N., Huang I.-C. (2024). Using natural language processing to analyze unstructured patient-reported outcomes data derived from electronic health records for cancer populations: A systematic review. Expert Rev. Pharmacoeconomics Outcomes Res..

[24] Vyas A., Kumar K., Sharma A., Verma D., Bhatia D., Wahi N., Yadav A.K. (2025). Advancing the frontier of artificial intelligence on emerging technologies to redefine cancer diagnosis and care. Comput. Biol. Med..

[25] Pecorino L. (2021). Molecular Biology of Cancer: Mechanisms, Targets, and Therapeutics.

[26] Hamamoto R., Suvarna K., Yamada M., Kobayashi K., Shinkai N., Miyake M., Takahashi M., Jinnai S., Shimoyama R., Sakai A. (2020). Application of artificial intelligence technology in oncology: Towards the establishment of precision medicine. Cancers.

[27] Rehan H. (2024). Advancing cancer treatment with AI-driven personalized medicine and cloud-based data integration. J. Mach. Learn. Pharma-Ceutical Res..

[28] Marchiano R.D.M., Di Sante G., Piro G., Carbone C., Tortora G., Boldrini L., Pietragalla A., Daniele G., Tredicine M., Cesario A. (2021). Translational research in the era of precision medicine: Where we are and where we will go. J. Pers. Med..

[29] Elemento O., Leslie C., Lundin J., Tourassi G. (2021). Artificial intelligence in cancer research, diagnosis and therapy. Nat. Rev. Cancer.

[30] Bhinder B., Gilvary C., Madhukar N.S., Elemento O. (2021). Artificial intelligence in cancer research and precision medicine. Cancer Discov..

[31] Liao J., Li X., Gan Y., Han S., Rong P., Wang W., Li W., Zhou L. (2023). Artificial intelligence assists precision medicine in cancer treatment. Front. Oncol..

[32] Luchini C., Pea A., Scarpa A. (2022). Artificial intelligence in oncology: Current applications and future perspectives. Br. J. Cancer.

[33] Hunter B., Hindocha S., Lee R.W. (2022). The role of artificial intelligence in early cancer diagnosis. Cancers.

[34] Bi W.L., Hosny A., Schabath M.B., Giger M.L., Birkbak N.J., Mehrtash A., Allison T., Arnaout O., Abbosh C., Dunn I.F. (2019). Artificial intelligence in cancer imaging: Clinical challenges and applications. CA A Cancer J. Clin..

[35] Perez-Lopez R., Ghaffari Laleh N., Mahmood F., Kather J.N. (2024). A guide to artificial intelligence for cancer researchers. Nat. Rev. Cancer.

[36] Murmu A., Győrffy B. (2024). Artificial intelligence methods available for cancer research. Front. Med..

[37] Corti C., Cobanaj M., Dee E.C., Criscitiello C., Tolaney S.M., Celi L.A., Curigliano G. (2023). Artificial intelligence in cancer research and precision medicine: Applications, limitations and priorities to drive transformation in the delivery of equitable and unbiased care. Cancer Treat. Rev..

[38] Prelaj A., Miskovic V., Zanitti M., Trovo F., Genova C., Viscardi G., Rebuzzi S., Mazzeo L., Provenzano L., Kosta S. (2024). Artificial intelligence for predictive biomarker discovery in immuno-oncology: A systematic review. Ann. Oncol..

[39] Shmatko A., Ghaffari Laleh N., Gerstung M., Kather J.N. (2022). Artificial intelligence in histopathology: Enhancing cancer research and clinical oncology. Nat. Cancer.

[40] Mao Y., Shangguan D., Huang Q., Xiao L., Cao D., Zhou H., Wang Y.-K. (2025). Emerging artificial intelligence-driven precision therapies in tumor drug resistance: Recent advances, opportunities, and challenges. Mol. Cancer.

[41] Chang T.-G., Park S., Schäffer A.A., Jiang P., Ruppin E. (2025). Hallmarks of artificial intelligence contributions to precision oncology. Nat. Cancer.

[42] Dlamini Z., Francies F.Z., Hull R., Marima R. (2020). Artificial intelligence (AI) and big data in cancer and precision oncology. Comput. Struct. Biotechnol. J..

[43] He X., Liu X., Zuo F., Shi H., Jing J. (2023). Artificial intelligence-based multi-omics analysis fuels cancer precision medicine. Semin. Cancer Biol..

[44] Patel S.K., George B., Rai V. (2020). Artificial intelligence to decode cancer mechanism: Beyond patient stratification for precision oncology. Front. Pharmacol..

[45] Alvarez-Torres M.d.M., Fu X., Rabadan R. (2025). Illuminating the dark genome in cancer using artificial intelligence. Cancer Res..

[46] Chadha S., Mukherjee S., Sanyal S. (2025). Advancements and implications of artificial intelligence for early detection, diagnosis and tailored treatment of cancer. Semin. Oncol..

[47] Li Q., Geng S., Luo H., Wang W., Mo Y.-Q., Luo Q., Wang L., Song G.-B., Sheng J.-P., Xu B. (2024). Signaling pathways involved in colorectal cancer: Pathogenesis and targeted therapy. Signal Transduct. Target. Ther..

[48] Niu Z., Jin R., Zhang Y., Li H. (2022). Signaling pathways and targeted therapies in lung squamous cell carcinoma: Mechanisms and clinical trials. Signal Transduct. Target. Ther..

[49] Wang J., Zhang Z., Wang Y. (2025). Utilizing feature selection techniques for AI-driven tumor subtype classification: Enhancing precision in cancer diagnostics. Biomolecules.

[50] Wang R.C., Wang Z. (2023). Precision medicine: Disease subtyping and tailored treatment. Cancers.

[51] Arzikulov F., Komiljonov A. (2025). The role of artificial intelligence in personalized oncology: Predictive models and treatment optimization. Acad. J. Sci. Technol. Educ..

[52] Yates J., Van Allen E.M. (2025). New horizons at the interface of artificial intelligence and translational cancer research. Cancer Cell.

[53] Luan W.-Y., Zhao Q., Zhang Z., Xu Z.-X., Lin S.-X., Miao Y.-D. (2025). Multidimensional decoding of colorectal cancer heterogeneity: Artificial intelligence-enabled precision exploration of single-cell and spatial transcriptomics. World J. Gastrointest. Oncol..

[54] Shin H., Lee D., Kim Y., Lee D., Na K.J., Cha C.D., Park H., Choi H. (2025). A self-supervised AI model leveraging spatial omics for analyzing tumor microenvironment heterogeneity in breast cancer only with H&E. Cancer Res..

[55] Martínez-García M., Hernández-Lemus E. (2022). Data integration challenges for machine learning in precision medicine. Front. Med..

[56] Li J., Li L., You P., Wei Y., Xu B. (2023). Towards artificial intelligence to multi-omics characterization of tumor heterogeneity in esophageal cancer. Semin. Cancer Biol..

[57] Jha M., Hasija Y. (2025). Development and validation of AI-driven multi-omics language models for cancer genomics: A comprehensive review. Comput. Biol. Chem..

[58] Li L., Sun M., Wang J., Wan S. (2024). Multi-omics based artificial intelligence for cancer research. Adv. Cancer Res..

[59] Saxena S., Jena B., Gupta N., Das S., Sarmah D., Bhattacharya P., Nath T., Paul S., Fouda M.M., Kalra M. (2022). Role of artificial intelligence in radiogenomics for cancers in the era of precision medicine. Cancers.

[60] Xu L., Saunders K., Huang S.-P., Knutsdottir H., Martinez-Algarin K., Terrazas I., Chen K., McArthur H.M., Maués J., Hodgdon C. (2024). A comprehensive single-cell breast tumor atlas defines epithelial and immune heterogeneity and interactions predicting anti-PD-1 therapy response. Cell Rep. Med..

[61] Shulman E.D., Campagnolo E.M., Lodha R., Stemmer A., Cantore T., Hu T., Nasrallahm M., Hoang D.-T., Aldape K., Ruppin K. (2024). Path2Space: An AI approach for cancer biomarker discovery via histopathology inferred spatial transcriptomics. bioRxiv.

[62] Jiang L., You C., Xiao Y., Wang H., Su G.-H., Xia B.-Q., Zheng R.-C., Zhang D.-D., Jiang Y.-Z., Gu Y.-J. (2022). Radiogenomic analysis reveals tumor heterogeneity of triple-negative breast cancer. Cell Rep. Med..

[63] Rabuzin L. (2024). Graph Neural Network-Driven Analysis of Cellular Organization for Non-Small Cell Lung Cancer Precision Oncology. Master’s Thesis.

[64] Liu T., Fang Z., Li X., Zhang L., Cao D.-S., Li M., Yin M. (2024). Assembling spatial clustering framework for heterogeneous spatial transcriptomics data with GRAPHDeep. Bioinformatics.

[65] Hu Y., Li X., Yi Y., Huang Y., Wang G., Wang D. (2025). Deep learning-driven survival prediction in pan-cancer studies by integrating multimodal histology-genomic data. Brief. Bioinform..

[66] Mann M., Kumar C., Zeng W.-F., Strauss M.T. (2021). Artificial intelligence for proteomics and biomarker discovery. Cell Syst..

[67] Reel P.S., Reel S., Pearson E., Trucco E., Jefferson E. (2021). Using machine learning approaches for multi-omics data analysis: A review. Biotechnol. Adv..

[68] Ali H. (2023). Artificial intelligence in multi-omics data integration: Advancing precision medicine, biomarker discovery and genomic-driven disease interventions. Int. J. Sci. Res. Arch..

[69] Wei L., Niraula D., Gates E.D.H., Fu J., Luo Y., Nyflot M.J., Bowen S.R., El Naqa I.M., Cui S. (2023). Artificial intelligence (AI) and machine learning (ML) in precision oncology: A review on enhancing discoverability through multiomics integration. Br. J. Radiol..

[70] Srivastava R. (2025). Advancing precision oncology with AI-powered genomic analysis. Front. Pharmacol..

[71] Gaetz J., Wall T.R., Stylianou E.S., Khalil E., Levy O., Selinger D.W. (2025). Abstract A019: A fully transparent and automatable form of AI for biomarker and new target discovery using diverse multi-omics data. Clin. Cancer Res..

[72] Boehm K.M., Khosravi P., Vanguri R., Gao J., Shah S.P. (2022). Harnessing multimodal data integration to advance precision oncology. Nat. Rev. Cancer.

[73] DeGroat W., Abdelhalim H., Peker E., Sheth N., Narayanan R., Zeeshan S., Liang B.T., Ahmed Z. (2024). Multimodal AI/ML for discovering novel biomarkers and predicting disease using multi-omics profiles of patients with cardiovascular diseases. Sci. Rep..

[74] Lan W., Liao H., Chen Q., Zhu L., Pan Y., Chen Y.-P.P. (2024). DeepKEGG: A multi-omics data integration framework with biological insights for cancer recurrence prediction and biomarker discovery. Brief. Bioinform..

[75] Pun F.W., Ozerov I.V., Zhavoronkov A. (2023). AI-powered therapeutic target discovery. Trends Pharmacol. Sci..

[76] Choi Y., Kim S., Kim Y., Song Y., Jang J., Donnelly D., Rajput B., Liu T.-L., Baek K., Sathe A. (2025). Discovery of potential synergistic targets for PKMYT1 inhibitor through AI-assisted drug combination screening in an MPS-based vascularized metastatic gastric cancer model. Cancer Res..

[77] Soenens A., Miller D., Vipond O., Skuras F., Brennan A., Hercot A. (2025). AI driven discovery of novel synthetic lethal gene pairs for targeted cancer therapy. Cancer Res..

[78] Elgawish M.S., Almatary A.M., Zaitone S.A., Salem M.S. (2025). Leveraging artificial intelligence and machine learning in kinase inhibitor development: Advances, challenges, and future prospects. RSC Med. Chem..

[79] Peng Z., Ding Y., Zhang P., Lv X., Li Z., Zhou X., Huang S. (2024). Artificial intelligence application for anti-tumor drug synergy prediction. Curr. Med. Chem..

[80] Liu T., Zhong L., Sun X., He Z., Lv W., Deng L., Chen Y. (2025). Machine learning-driven multi-targeted drug discovery in colon cancer using biomarker signatures. Precis. Oncol..

[81] Abbas M., Rassam A., Karamshahi F., Abunora R., Abouseada M. (2024). The role of AI in drug discovery. ChemBioChem.

[82] Qureshi R., Irfan M., Gondal T.M., Khan S., Wu J., Hadi M.U., Heymach J., Le X., Yan H., Alam T. (2023). AI in drug discovery and its clinical relevance. Heliyon.

[83] Serrano D.R., Luciano F.C., Anaya B.J., Ongoren B., Kara A., Molina G., Ramirez B.I., Sánchez-Guirales S.A., Simon J.A., Tomietto G. (2024). Artificial intelligence (AI) applications in drug discovery and drug delivery: Revolutionizing personalized medicine. Pharmaceutics.

[84] Ocana A., Pandiella A., Privat C., Bravo I., Luengo-Oroz M., Amir E., Gyorffy B. (2025). Integrating artificial intelligence in drug discovery and early drug development: A transformative approach. Biomark. Res..

[85] Yang Y., Cheng F. (2025). Artificial intelligence streamlines scientific discovery of drug–target interactions. Br. J. Pharmacol..

[86] Vora N., Shah S., Patel P., Shah M. (2025). Artificial intelligence and multi-omics in drug discovery: A deep learning-powered revolution. Cure Care.

[87] Korshunova M., Ginsburg B., Tropsha A., Isayev O. (2021). OpenChem: A deep learning toolkit for computational chemistry and drug design. J. Chem. Inf. Model..

[88] Paul K., Gowda B.J. (2025). Artificial Intelligence in Drug Repurposing: Revolutionizing Drug Discovery through Computational Case Studies. Biopress J. Comput. Life Sci..

[89] Jain P., Jain S.K., Jain M. (2021). Harnessing drug repurposing for exploration of new diseases: An insight to strategies and case studies. Curr. Mol. Med..

[90] Gupta R., Srivastava D., Sahu M., Tiwari S., Ambasta R.K., Kumar P. (2021). Artificial intelligence to deep learning: Machine intelligence approach for drug discovery. Mol. Divers..

[91] Dermawan D., Alotaiq N. (2025). From lab to clinic: How artificial intelligence (AI) is reshaping drug discovery timelines and industry outcomes. Pharmaceuticals.

[92] Kucukzeybek B.B., Dere Y., Sari A.A., Ocal I., Avcu E., Dere O., Calli A.O., Dinckal C., Tunakan M., Kucukzeybek Y. (2024). The prognostic significance of CD117-positive mast cells and microvessel density in colorectal cancer. Medicine.

[93] Blanco-González A., Cabezón A., Seco-González A., Conde-Torres D., Antelo-Riveiro P., Piñeiro Á., Garcia-Fandino R. (2023). The role of AI in drug discovery: Challenges, opportunities, and strategies. Pharmaceuticals.

[94] Kolluri S., Lin J., Liu R., Zhang Y., Zhang W. (2022). Machine Learning and Artificial Intelligence in Pharmaceutical Research and Development: A Review: Machine Learning and Artificial Intelligence in Pharmaceutical R&D. AAPS J..

[95] Yang X., Wang Y., Byrne R., Schneider G., Yang S. (2019). Concepts of artificial intelligence for computer-assisted drug discovery. Chem. Rev..

[96] Deng F., Feng C.H., Gao N., Zhang L. (2025). Normalization and selecting non-differentially expressed genes improve machine learning modelling of cross-platform transcriptomic data. Trans. Artif. Intell..

[97] Wei F., Kouro T., Nakamura Y., Ueda H., Iiizumi S., Hasegawa K., Asahina Y., Kishida T., Morinaga S., Himuro H. (2024). Enhancing Mass spectrometry-based tumor immunopeptide identification: Machine learning filter leveraging HLA binding affinity, aliphatic index and retention time deviation. Comput. Struct. Biotechnol. J..

[98] Han R., Yoon H., Kim G., Lee H., Lee Y. (2023). Revolutionizing medicinal chemistry: The application of artificial intelligence (AI) in early drug discovery. Pharmaceuticals.

[99] Gangwal A., Lavecchia A. (2024). Unleashing the power of generative AI in drug discovery. Drug Discov. Today.

[100] Malheiro V., Santos B., Figueiras A., Mascarenhas-Melo F. (2025). The potential of artificial intelligence in pharmaceutical innovation: From drug discovery to clinical trials. Pharmaceuticals.

[101] Walters W.P., Murcko M. (2020). Assessing the impact of generative AI on medicinal chemistry. Nat. Biotechnol..

[102] Garg P., Singhal G., Kulkarni P., Horne D., Salgia R., Singhal S.S. (2024). Artificial intelligence–driven computational approaches in the development of anticancer drugs. Cancers.

[103] Duo L., Liu Y., Ren J., Tang B., Hirst J.D. (2024). Artificial intelligence for small molecule anticancer drug discovery. Expert Opin. Drug Discov..

[104] Poyatos-Racionero E., Paniagua-Herranz L., Privat C., Martín-Hernández C., Nieto-Jiménez C., Herrero-Igartua C., García-Escudero R., Gancarski P., Lorz C., Ocana A. (2025). Validation of an AI-powered computational chemistry workflow for streamlined drug discovery. Mol. Cancer Ther..

[105] Zhang S. (2026). Abstract C008: AlphaFold Beyond Folding: Protein Property Prediction and Drug Discovery. Ph.D. Thesis.

[106] Wang S., Böhnert V., Joseph A.J., Sudaryo V., Skariah G., Swinderman J.T., Yu F.B., Subramanyam V., Wolf D.M., Lyu X. (2023). ENPP1 is an innate immune checkpoint of the anticancer cGAMP–STING pathway in breast cancer. Proc. Natl. Acad. Sci. USA.

[107] Ren S., Cooper G.F., Chen L., Lu X. (2024). An interpretable deep learning framework for genome-informed precision oncology. Nat. Mach. Intell..

[108] Wu Y., Chen M., Qin Y. (2025). Anticancer drug response prediction integrating multi-omics pathway-based difference features and multiple deep learning techniques. PLoS Comput. Biol..

[109] Cellina M., Cè M., Khenkina N., Sinichich P., Cervelli M., Poggi V., Boemi S., Ierardi A.M., Carrafiello G. (2022). Artificial intelligence in the era of precision oncological imaging. Technol. Cancer Res. Treat..

[110] Li L., Doppalapudi A., Escamilla J., Karithara A., Pham C., Phillip A., Binoy A., Gullapalli S., Baldado L., Bellamkonda A. (2025). Precision medicine and beyond: Evolving roles of targeted therapy, immunotherapy, and artificial intelligence in oncology. INNOSC Theranostics Pharmacol. Sci..

[111] Bouriga R., Bailleux C., Gal J., Chamorey E., Mograbi B., Hannoun-Levi J.-M., Milano G. (2025). Advances and critical aspects in cancer treatment development using digital twins. Brief. Bioinform..

[112] Shen S., Qi W., Liu X., Zeng J., Li S., Zhu X., Dong C., Wang B., Shi Y., Yao J. (2025). From virtual to reality: Innovative practices of digital twins in tumor therapy. J. Transl. Med..

[113] Stahlberg E.A., Abdel-Rahman M., Aguilar B., Asadpoure A., Beckman R.A., Borkon L.L., Bryan J.N., Cebulla C.M., Chang Y.H., Chatterjee A. (2022). Exploring approaches for predictive cancer patient digital twins: Opportunities for collaboration and innovation. Front. Digit. Health.

[114] D’orsi L., Capasso B., Lamacchia G., Pizzichini P., Ferranti S., Liverani A., Fontana C., Panunzi S., De Gaetano A., Presti E.L. (2024). Recent advances in artificial intelligence to improve immunotherapy and the use of digital twins to identify prognosis of patients with solid tumors. Int. J. Mol. Sci..

[115] Jin H., Wang L., Bernards R. (2023). Rational combinations of targeted cancer therapies: Background, advances and challenges. Nat. Rev. Drug Discov..

[116] Morales-Durán N., León-Buitimea A., Morones-Ramírez J.R. (2024). Unraveling resistance mechanisms in combination therapy: A comprehensive review of recent advances and future directions. Heliyon.

[117] Hilal T., Gonzalez-Velez M., Prasad V. (2020). Limitations in clinical trials leading to anticancer drug approvals by the US Food and Drug Administration. JAMA Intern. Med..

[118] Harkos C., Hadjigeorgiou A.G., Voutouri C., Kumar A.S., Stylianopoulos T., Jain R.K. (2025). Using mathematical modelling and AI to improve delivery and efficacy of therapies in cancer. Nat. Rev. Cancer.

[119] Mathur D., Barnett E., Scher H.I., Xavier J.B. (2022). Optimizing the future: How mathematical models inform treatment schedules for cancer. Trends Cancer.

[120] Horgan D., Baird A.-M., Middleton M., Mihaylova Z., Van Meerbeeck J.P., Vogel-Claussen J., Van Schil P.E., Malvehy J., Ascierto P.A., Dube F. (2022). How can the EU beating cancer plan help in tackling lung cancer, colorectal cancer, breast cancer and melanoma?. Healthcare.

[121] Sherani A.M.K., Khan M., Qayyum M.U., Hussain H.K. (2024). Synergizing AI and healthcare: Pioneering advances in cancer medicine for personalized treatment. Int. J. Multidiscip. Sci. Arts.

[122] Chehelgerdi M., Chehelgerdi M., Khorramian-Ghahfarokhi M., Shafieizadeh M., Mahmoudi E., Eskandari F., Rashidi M., Arshi A., Mokhtari-Farsani A. (2024). Comprehensive review of CRISPR-based gene editing: Mechanisms, challenges, and applications in cancer therapy. Mol. Cancer.

[123] Li M., Sun J., Shi G. (2023). Application of CRISPR screen in mechanistic studies of tumor development, tumor drug resistance, and tumor immunotherapy. Front. Cell Dev. Biol..

[124] AlDoughaim M., AlSuhebany N., AlZahrani M., AlQahtani T., AlGhamdi S., Badreldin H., Al Alshaykh H. (2024). Cancer biomarkers and precision oncology: A review of recent trends and innovations. Clin. Med. Insights Oncol..

[125] Cockburn J.G., Mariappan V., Loke M.F., Al-Maleki A.R., Muttiah B., Vellasamy K.M., Vadivelu J. (2025). Integrative research: Current trends and considerations for biomarker discovery and precision medicine. Microbe.

[126] Aktar N., Yueting C., Abbas M., Zafar H., Paiva-Santos A.C., Zhang Q., Chen T., Ahmed M., Raza F., Zhou X. (2022). Understanding of immune escape mechanisms and advances in cancer immunotherapy. J. Oncol..

[127] Noorbakhsh J., Foroughi Pour A., Chuang J. (2025). Emerging AI approaches for cancer spatial omics. GigaScience.

[128] Sarkar C., Das B., Rawat V.S., Wahlang J.B., Nongpiur A., Tiewsoh I., Lyngdoh N.M., Das D., Bidarolli M., Sony H.T. (2023). Artificial intelligence and machine learning technology driven modern drug discovery and development. Int. J. Mol. Sci..

[129] Saraf S., De A., Tripathy B. (2024). Effective use of computational biology and artificial intelligence in the domain of medical oncology. Computational Intelligence for Oncology and Neurological Disorders.

[130] Dankwa-Mullan I., Ndoh K., Akogo D., Rocha H.A.L., Juaçaba S.F. (2025). Artificial intelligence and cancer health equity: Bridging the divide or widening the gap. Curr. Oncol. Rep..

[131] Smiley A., Reategui-Rivera C.M., Villarreal-Zegarra D., Escobar-Agreda S., Finkelstein J. (2025). Exploring artificial intelligence biases in predictive models for cancer diagnosis. Cancers.

[132] Hantel A., Walsh T.P., Marron J.M., Kehl K.L., Sharp R., Van Allen E., Abel G.A. (2024). Perspectives of oncologists on the ethical implications of using artificial intelligence for cancer care. JAMA Netw. Open.

[133] Froicu E.-M., Creangă-Murariu I., Afrăsânie V.-A., Gafton B., Alexa-Stratulat T., Miron L., Pușcașu D.M., Poroch V., Bacoanu G., Radu I. (2025). Artificial intelligence and decision-making in oncology: A review of ethical, legal, and informed consent challenges. Curr. Oncol. Rep..

[134] Du Z., Lian L., Xi W., Zheng Y., Yu G., Luo H.-Y., Qin P. (2025). Artificial intelligence enables the ethical reconstruction and social value realization of global cancer research: From technological innovation to humanistic care. Clin. Cancer Res..

[135] Cheng C.H., Shi S.-S. (2025). Artificial intelligence in cancer: Applications, challenges, and future perspectives. Mol. Cancer.

[136] Chamouni G., Lococo F., Sassorossi C., Atuhaire N., Ádány R., Varga O. (2025). Ethical and legal concerns in artificial intelligence applications for the diagnosis and treatment of lung cancer: A scoping review. Front. Public Health.

